# Comparative Gene Expression Profiles Induced by PPARγ and PPARα/γ Agonists in Human Hepatocytes

**DOI:** 10.1371/journal.pone.0018816

**Published:** 2011-04-18

**Authors:** Alexandra Rogue, Carine Lambert, Rozenn Jossé, Sebastien Antherieu, Catherine Spire, Nancy Claude, André Guillouzo

**Affiliations:** 1 UMR INSERM U991, Faculté des Sciences Pharmaceutiques et Biologiques, Rennes, France; 2 Université de Rennes 1, Rennes, France; 3 Biologie Servier, Gidy, France; 4 Institut de Recherches Servier, Courbevoie, France; Florida International University, United States of America

## Abstract

**Background:**

Several glitazones (PPARγ agonists) and glitazars (dual PPARα/γ agonists) have been developed to treat hyperglycemia and, simultaneously, hyperglycemia and dyslipidemia, respectively. However, most have caused idiosyncratic hepatic or extrahepatic toxicities through mechanisms that remain largely unknown. Since the liver plays a key role in lipid metabolism, we analyzed changes in gene expression profiles induced by these two types of PPAR agonists in human hepatocytes.

**Methodology/Principal Findings:**

Primary human hepatocytes and the well-differentiated human hepatoma HepaRG cells were exposed to different concentrations of two PPARγ (troglitazone and rosiglitazone) and two PPARα/γ (muraglitazar and tesaglitazar) agonists for 24 h and their transcriptomes were analyzed using human pangenomic Agilent microarrays. Principal Component Analysis, hierarchical clustering and Ingenuity Pathway Analysis® revealed large inter-individual variability in the response of the human hepatocyte populations to the different compounds. Many genes involved in lipid, carbohydrate, xenobiotic and cholesterol metabolism, as well as inflammation and immunity, were regulated by both PPARγ and PPARα/γ agonists in at least a number of human hepatocyte populations and/or HepaRG cells. Only a few genes were selectively deregulated by glitazars when compared to glitazones, indicating that PPARγ and PPARα/γ agonists share most of their target genes. Moreover, some target genes thought to be regulated only in mouse or to be expressed in Kupffer cells were also found to be responsive in human hepatocytes and HepaRG cells.

**Conclusions/Significance:**

This first comprehensive analysis of gene regulation by PPARγ and PPARα/γ agonists favor the conclusion that glitazones and glitazars share most of their target genes and induce large differential changes in gene profiles in human hepatocytes depending on hepatocyte donor, the compound class and/or individual compound, thereby supporting the occurrence of idiosyncratic toxicity in some patients.

## Introduction

Peroxisome proliferator-activated receptors (PPARs) are an important class of ligand-activated transcription factors involved in the regulation of nutrient homeostasis, as well as a variety of other biological processes [1]. This superfamily of nuclear receptors comprises 3 subtypes: PPARα, PPARβ/δ and PPARγ, also known as NR1C1, NR1C2 and NR1C3, respectively [2]. Synthetic drugs activating PPARα and PPARγ are in clinical use: the former typified by fibrates, are used to treat dyslipidemia, while the latter include glitazones that act as insulin sensitizers in type 2 diabetes mellitus [3]. First generation of glitazones were found to be highly hepatotoxic: the first one, ciglitazone, was abandoned after clinical trials and the second, troglitazone (TRO), was rapidly withdrawn from the market after reports of severe liver failure and death [4]. By contrast, the second generation of glitazones developed as PPARγ agonists, namely rosiglitazone (ROSI) and pioglitazone, have been shown to cause much less frequent and severe hepatotoxicity. Dual PPARα and PPARγ agonists have also been developed by the pharmaceutical industry for the simultaneous treatment of hyperglycemia and dyslipidemia, but the first developed drugs, muraglitazar (MURA) and tesaglitazar (TESA), were terminated during clinical trials due to cardiac and renal side-effects, despite the absence of noticeable hepatic lesions [5]. The mechanisms of these idiosyncratic toxicities of glitazones and glitazars in humans remain unclear.

Major species-differences have been observed in liver sensitivity to PPAR agonists as first witnessed with fibrates, which have safely been used for years to lower plasma triglycerides in humans, whereas in rodents they induced various hepatic lesions, including increased peroxisome proliferation in addition to hepatic hypertrophy and hyperplasia that ultimately result in liver tumors [6], [7]. Preclinical animal studies did not predict glitazone hepatotoxicity or glitazar cardiac and renal toxicities in humans. Therefore, it might be postulated that both glitazones and glitazars regulate different sets of genes in humans and rodents. Consequently, human liver cell models should represent a more appropriate approach than their rodent counterparts for investigations of the hepatotoxic effects of PPAR agonists. In spite of limitations due to scarce availability, interindividual variability and short-term *in vitro* life-span that does not allow the study of long term effects of chemicals, primary human hepatocyte cultures are recognized as the most appropriate *in vitro* system for investigations of drug-induced hepatic effects [8].

To our knowledge, a comprehensive analysis of gene regulation by PPARγ and PPARα/γ agonists in human hepatocytes has not been published. The aim of the present study was to identify changes in gene expression profiles induced by PPARγ and PPARα/γ agonists in human hepatocytes from several donors and in differentiated human hepatoma HepaRG cells using a whole genome transcriptomic approach. The HepaRG cell line represents a potentially suitable surrogate to primary hepatocytes since it combines the advantages of the expression of most of the liver-specific functions, including the major cytochromes P450 at levels comparable to those found in primary human hepatocytes and the relative functional stability for several weeks at confluence [9], [10].

Large inter-individual variations in gene expression profiles were highlighted in response to 24 h treatments with different PPARγ and PPARα/γ agonists. However, in addition to many common altered genes that have also frequently been identified as PPARα target genes, small subsets of genes were found to be restricted to either individual or one class of test agonists.

## Materials and Methods

### Chemicals

Williams' E medium was supplied by Eurobio (Les Ulis, France) and fetal calf serum (FCS) by Perbio (Brebieres, France). TRO, ROSI, MURA and TESA were synthesized by the Servier Chemical Department. [^3^H(G)]Taurocholic acid (sp. Act. 1.19 Ci/mmol) was purchased from Perkin-Elmer Life Science (Boston, MA) and dichlorofluorescein diacetate (DCFDA) was purchased from Invitrogen (Cergy Pontoise, France). All other chemicals were of the highest quality available.

### Primary human hepatocytes (PHH)

Human hepatocytes from four adult donors undergoing resection for primary and secondary tumors were obtained by collagenase perfusion of histologically normal liver fragments [11] (Table S1). They were seeded at a density of 17×10^4^ cells/cm^2^ in 6-well dishes in a Williams E medium supplemented with 10% fetal calf serum (FCS), 100 units/µL penicillin, 100 µg/mL streptomycin, 1 µg/mL insulin, 2 mM glutamine and 1 µg/mL bovine serum albumin. The medium was discarded 12 h after seeding and cells were thereafter maintained in serum-free medium supplemented with 10^−7^ M hydrocortisone hemisuccinate.

### HepaRG cells

The HepaRG cell line is derived from a liver tumor of a female patient [12]. For the present studies, HepaRG cells were first seeded at a density of 2.6×10^4^ cells/cm^2^ in 6-well dishes in a Williams' E medium supplemented with 10% FCS, 100 units/mL penicillin, 100 µg/mL streptomycin, 5 µg/mL insulin, 2 mM glutamine and 5×10^−5^ M hydrocortisone hemisuccinate. After two weeks of culture, they were shifted to the same culture medium supplemented with 2% dimethylsulfoxide (DMSO) for two further weeks in order to reach maximum functional activities. Media were renewed every 2–3 days. Differentiated HepaRG cell cultures are composed of both hepatocyte-like and biliary-like cells (about 50% of each type) [13].

### PPAR agonist treatments

TRO, ROSI, MURA and TESA were dissolved in DMSO and stored frozen at a concentration of 300 mM until use. HepaRG cell cultures were exposed to varying concentrations of each compound for 24 h in FCS- and DMSO-free medium. Primary human hepatocytes were cultured for 24 h before exposure to the same concentrations of PPAR ligands.

### ATP assay

An ATP assay was used to estimate cell viability. ATP content was assayed in 96-well culture plates after 24 h exposure to 0, 5, 20, 40 µM of TRO; 0, 50, 100, 150 µM of ROSI and MURA and 0, 200, 300 and 2000 µM of TESA. At the end of the incubation period, cultures were observed under phase-contrast microscopy using an Olympus 1×70 microscope. Intracellular ATP content was measured using the Cell Titer-Glo Luminescent Cell Viability Assay kit (Promega, Charbonnières, France). ATP determinations were performed at least in triplicate. Results were normalized to control cells and expressed as mean ± SD.

### Measurement of caspase 3-like activity

After a 24 h treatment by PPAR agonists, differentiated HepaRG cells were harvested in the treatment medium and stored as pellets at − 80°C. After cell lysis, 40 µg of proteins were incubated with 80 µM Ac-DEVD-AMC in caspase-3 activity buffer (20 mM PIPES pH 7.2, 100 mM NaCl, 10 mM dithiotreitol, 1 mM EDTA, 0.1% CHAPS and 10% sucrose) at 37°C for 1 h. Caspase 3-mediated cleavage of Ac-DEVD-AMC peptide was continuously measured by spectrofluorimetry using excitation/emission wavelengths of 380/440 nm. The data were normalized to control values, and the control was expressed as a value of 100%.

### Determination of Reactive Oxygen Species (ROS) production

Cells were incubated in the dark at 37°C for 2 h with 0.33 µM DCFDA in culture medium. At the end of the incubation period, 300 µl of 5 ml of 200 µM K_2_HPO_4_/KH_2_PO_4_, 5 ml methanol and 10 µl triton X100 were added. The rate at which ROS formed the fluorescent product was measured with a microplate reader using excitation/emission wavelengths of 485/530 nm. The data were normalized to control values, and the control was expressed as a value of 100%.

### Western blotting

Fifty µg of total cellular protein extracts were resolved on 7.5% SDS–PAGE, transferred onto nitrocellulose membranes (Millipore, Guyancourt, France) and analyzed using chemiluminescence detection. The following antibodies were used: rabbit anti-human PPARγ (sc-29455, Santa Cruz biotechnology, Tebu, France) and mouse anti-human Heat Shock Cognate 70 (HSC70) (B-6, sc-7298, Santa Cruz Biotechnology, Tebu, France).

### RNA isolation

Cells were harvested in lysis buffer (RLT buffer and β-mercaptoethanol). Total RNA was isolated using the RNeasy mini Kit (Qiagen, Venlo, Netherlands). RNA quantity and purity were assessed with a Nanodrop ND-1000 spectrophotometer (Nyxor Biotech, Paris, France) and RNA integrity was checked on a Bioanalyzer 2100 (Agilent Technologies, Massy, France).

### Microarray hybridizations

Five hundred ng of total RNA from each control and PPAR agonist-treated cell culture were separately reverse-transcribed into double-strand cDNA by the Moloney murine leukaemia virus reverse transcriptase and amplified for 2 h at 40°C using Quick Amplification Labeling Kit (Agilent). The cDNA was then transcribed into antisense cRNA and labelled with either CTP-Cy3 or CTP-Cy5 fluorescent dyes for 2 h at 40°C following the manufacturer's protocol. Cyanine-labeled cRNAs were purified using RNeasy minikit (Qiagen). The cRNAs of both control and PPAR-treated HepaRG cells or PHH were hybridized on Agilent Gene chip human genome Microarrays (G4112F) according to standard Agilent protocols. Human hepatocyte and HepaRG cell samples were hybridized separately. Data analyses were performed using Rosetta Resolver v.7.0 software (Rosetta Biosoftware, Seattle, WA) for database management, quality control and analysis. All microarray data reported in this study complied with MIAME guidelines [14].

### qPCR analysis

Transcripts of some genes were also estimated by quantitative PCR in order to confirm microarrays results. Briefly, 500 ng of total RNA was reverse-transcribed into cDNA using the High-Capacity cDNA Archive kit (Applied Biosystems, Foster City, CA). qPCR was performed by the fluorescent dye SYBR Green methodology using the SYBR Green PCR Master Mix (Applied Biosystems) and the STEP one Plus (Applied Biosystems). Primer pairs for each transcript were chosen with qPrimer depot software (http://primerdepot.nci.nih.gov/) (Table S2). Amplification curves were read with the StepOne software V2.1 using the comparative cycle threshold method. The relative quantification of the steady-state mRNA levels was normalized against 18S mRNA.

### Sodium-taurocholate cotransporting polypeptide (NTCP) transport assays

Activity of the NTCP transporter was estimated by measuring sodium-dependent intracellular accumulation of radiolabeled taurocholate substrate as previously described [15]. Briefly, cells were incubated at 37°C for 30 min with 0.17 µM [^3^H]taurocholate in the presence or absence of sodium. After washing with phosphate-buffered saline, cells were lysed in 0.1 N NaOH, and accumulation of radiolabeled substrates was determined through scintillation counting. Taurocholate accumulation values in the presence minus absence of sodium represented NTCP activity.

### Statistical analysis

Normalization algorithms and background subtractions were automatically applied to each array to reduce systematic errors and to adjust effects due to technological rather than biological variations using Feature Extraction® and Resolver® software. Thereby, significantly modulated genes (at the Entrez Gene level) were analyzed according to a p-value ≤0.01 and a 1.5-fold change as filters. Principal component analysis (PCA) and hierarchical clustering were performed to visualize behaviour of data through cell models, products and concentrations. Biological functions and pathways were generated and analyzed using Ingenuity Pathway Analysis® v.7.0 (IPA, Ingenuity System, CA) from all data and relevant gene-sets. The Mann and Whitney test was used for statistical analysis of ATP, ROS, caspase 3 activity, PPARγ protein expression and NTCP activity values.

## Results

### Cytotoxic effects of PPAR agonists

**Figure 1:**
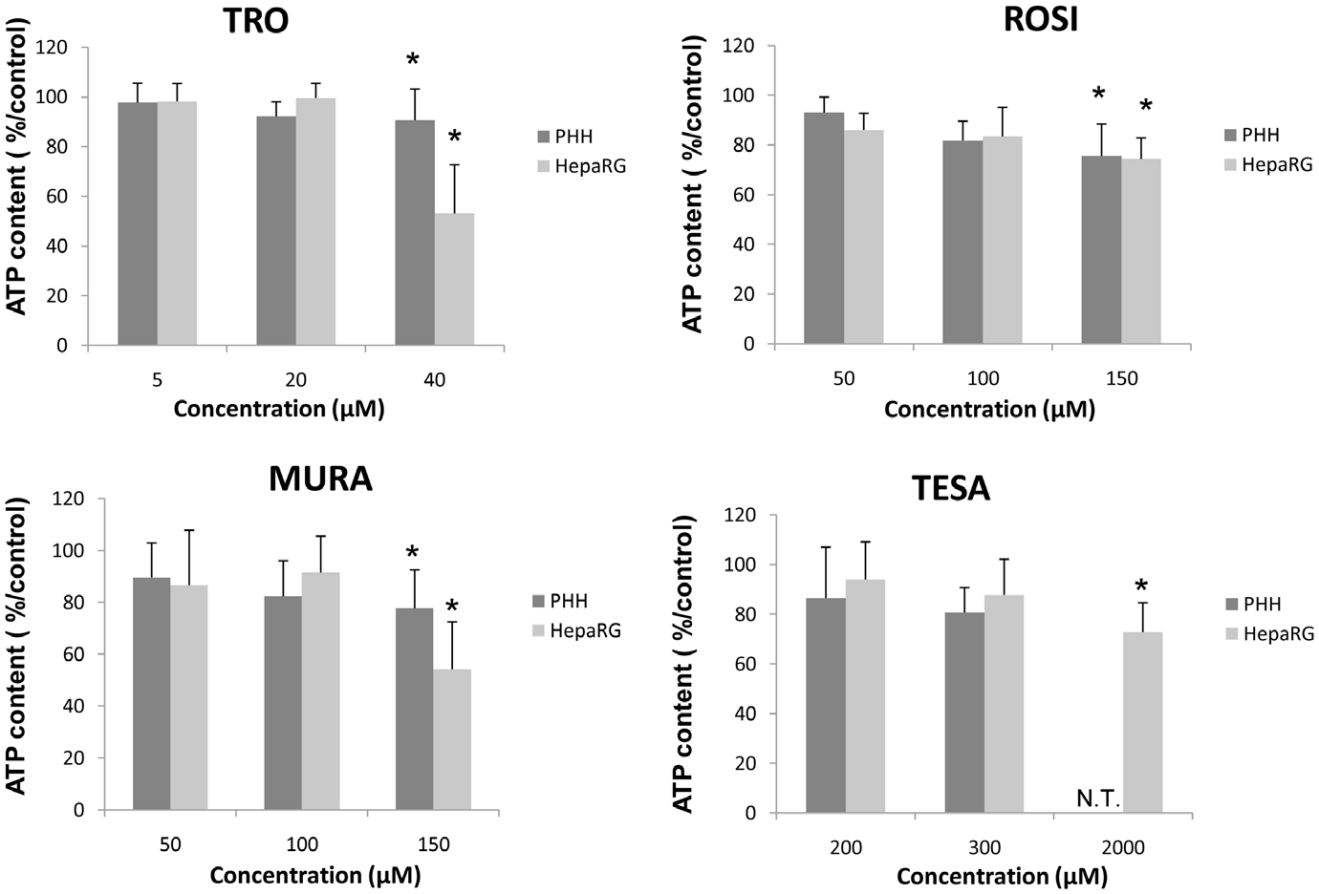
Intracellular ATP content in primary human hepatocytes and HepaRG cells treated with PPARγ or PPARα/γ agonists. Intracellular ATP content was measured in cells treated with TRO, ROSI, MURA or TESA for 24 h. Results are normalized to control cells and expressed as means ± S.D. of three independent experiments.* p<0.05, N.T.: non tested.

**Figure 2:**
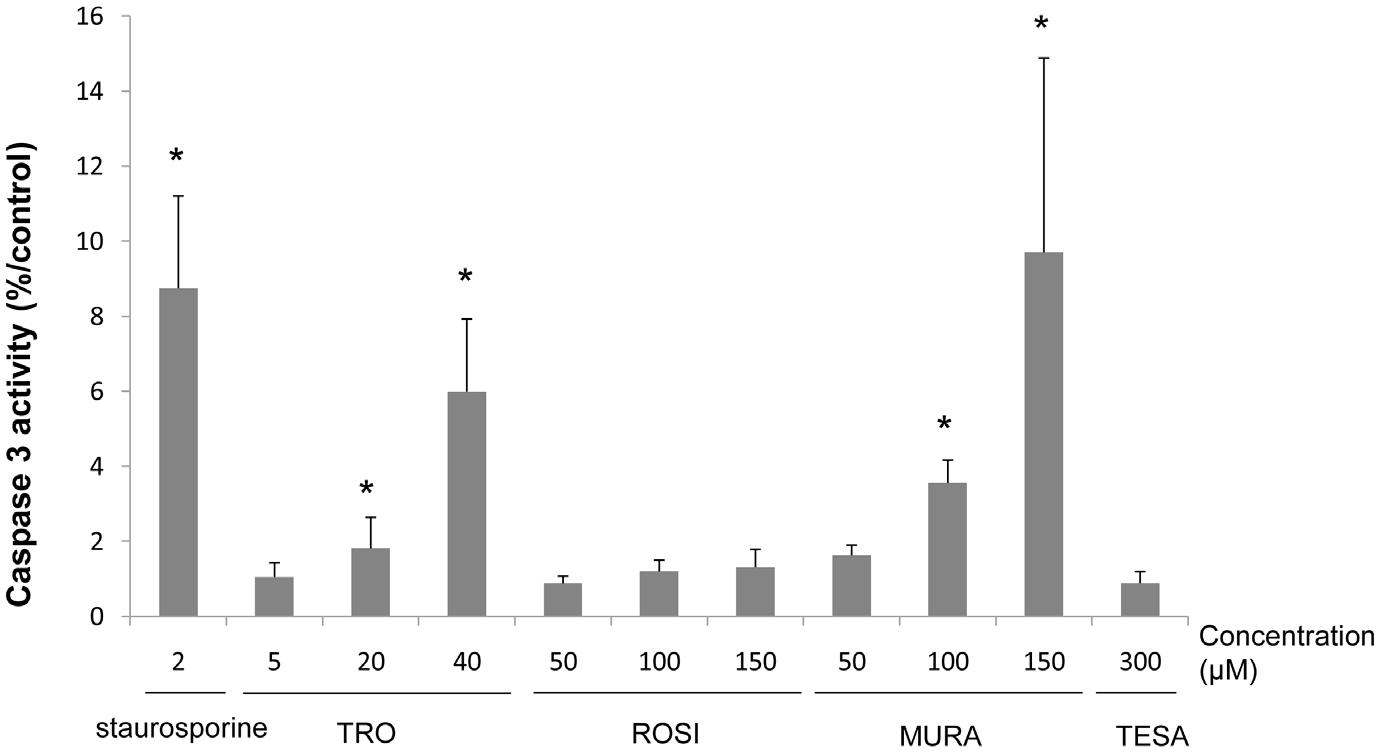
Caspase 3 activity in HepaRG cells treated with PPARγ or PPARα/γ agonists. Caspase 3 levels were determined in HepaRG cells after a 24 h treatment with TRO, ROSI, MURA or TESA. Staurosporine (2 µM) was used as a positive control. Results are normalized to control cells and expressed as means ± S.D. of three independent experiments.* p<0.05.

**Figure 3:**
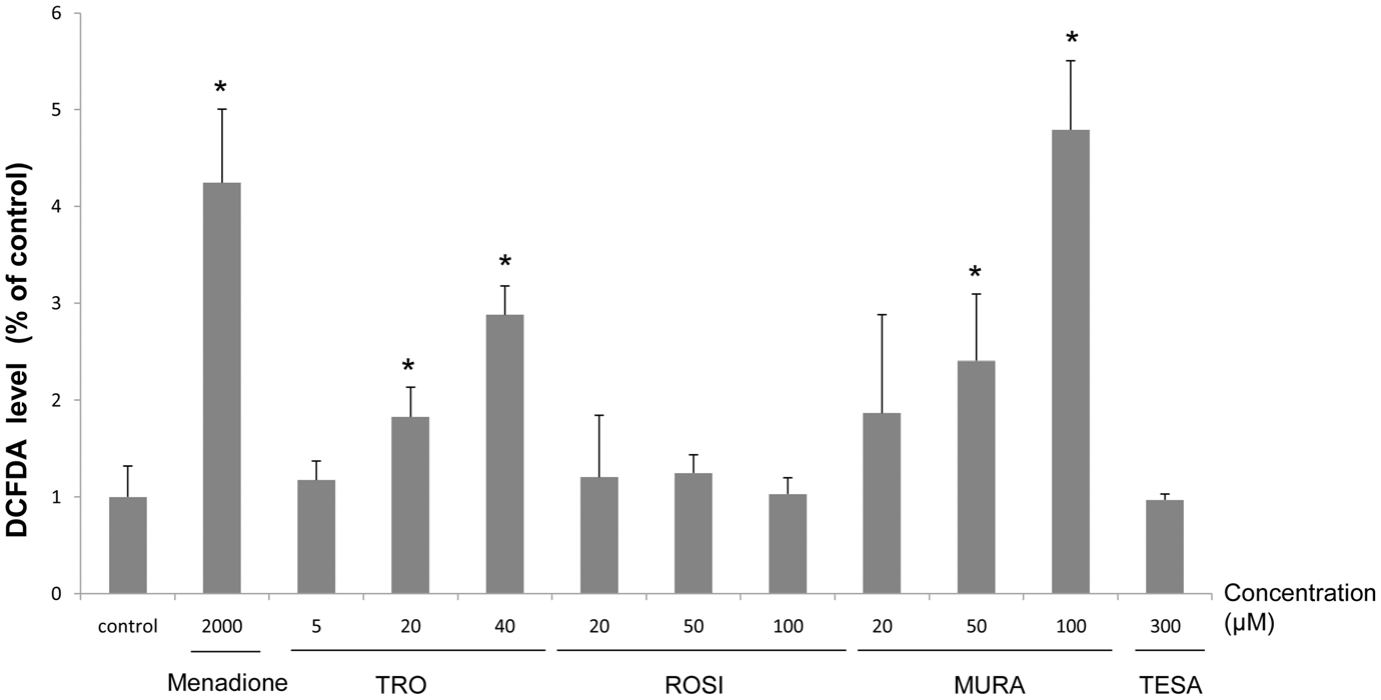
ROS levels in HepaRG cells treated by PPARγ or PPARα/γ agonists. ROS levels were estimated by measurement of intracellular DCFDA in HepaRG cells after a 24 h treatment by TRO, ROSI, MURA or TESA. Menadione (2 mM) was used as a positive control. Results are normalized to control cells and expressed as means ± S.D. of three independent experiments.* p<0.05.

Preliminary experiments were carried out to estimate the concentrations at which the four compounds caused cellular damage in primary human hepatocytes and HepaRG cells after a 24 h exposure, using the intracellular ATP content assay. TRO was ineffective at 5 and 20 µM in both cell models while at 40 µM it caused a greater decrease in ATP content in HepaRG cells, the values dropping to 53±19% (Figure 1). ROSI and MURA caused intracellular ATP changes from 150 µM, and TESA did not cause any effect, except a slight decrease at 2000 µM, in HepaRG cells. Examination of cell cultures at the end of the treatment time under phase-contrast microscopy revealed morphological alterations only in cultures exhibiting a significant decrease in ATP content. Toxic effects at the highest concentrations of TRO and MURA were confirmed by measuring caspase 3 activity (Figure 2) and an increase in ROS production was demonstrated by DCFDA quantification in HepaRG cells (Figure 3). 50 µM MURA, 100 µM MURA and 20 µM TRO induced a slightly but significantly increase of ROS production, caspase 3 activity and both respectively.

**Figure 4:**
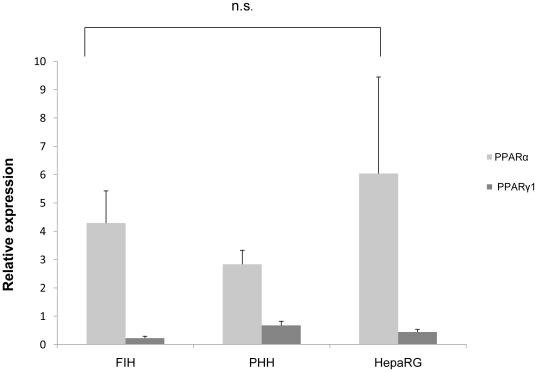
PPARα and PPARγ1 transcript levels in freshly isolated hepatocytes, primary human hepatocytes and HepaRG cells. Comparative expression of PPARα and PPARγ1 in freshly isolated hepatocytes (FIH), primary human hepatocytes (PHH) and differentiated HepaRG cells incubated in a medium containing 0.01% DMSO. The results are expressed relative to 18S and are the mean ± S.D.of at least three independent experiments.

**Figure 5:**
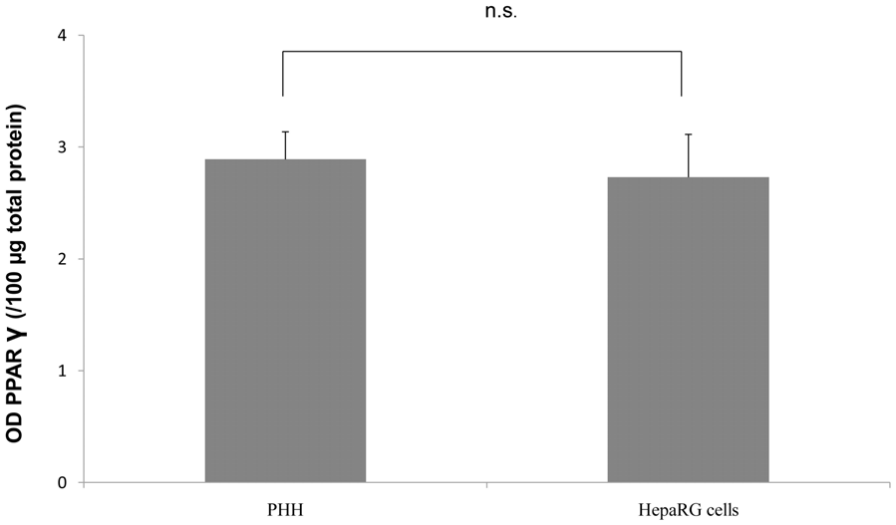
PPARγ protein level in primary human hepatocytes and HepaRG cells. Primary human hepatocytes and differentiated HepaRG cells were incubated in a medium containing 0.01% DMSO. The results are expressed as optical density per 100 µg total protein and are the mean ± S.D. of at least three independent experiments. ns, not statistically significant.

mRNA basal expression of PPARα and PPARγ was also measured by qPCR in freshly isolated human hepatocytes and in PHH and HepaRG cell cultures (Figure 4). No significant differences were observed in the three cell types. As expected, PPARα transcripts were found to be at least 3-fold more abundant than PPARγ transcripts. No expression of PPARγ2 was detected in any cell type (data not shown). Moreover, the comparable amount of PPARγ transcripts in the two cell culture models was confirmed at the protein level by western blotting analysis (Figure 5).

Based on all these initial data, a low, medium and subtoxic concentrations of TRO, ROSI and MURA were further selected for microarray analysis. Because of the absence of any cytotoxicity with TESA, even at 2000 µM, only one concentration of this PPAR agonist was studied.

### Numbers of deregulated genes

The numbers of total modulated genes, as well as the up- and down regulated genes in PHH and HepaRG cell cultures treated with each of the four compounds are displayed in Table 1. The numbers of modulated genes greatly varied depending on the hepatocyte donor, with a concentration-related effect in both cell models irrespective of the tested compound. Marked individual differences were seen in response to the PPAR agonists between the four human hepatocyte donors. Differences in gene expression with ROSI and MURA reached about 4 to 5-fold between the 4 donors at the low concentrations while they were only about 2-fold at high concentrations. Thus, at the lowest concentration tested, TRO modulated 428 to 2910 genes, ROSI 716 to 3135 genes, MURA 1642 to 3089 genes and TESA 2111 to 3277 genes. The lowest and the highest numbers of deregulated genes for each PPAR agonist were obtained in hepatocyte cultures from different donors. The lowest numbers of modulated genes with TRO, ROSI, MURA and TESA were observed with donors #3, #1, #2 and #2 respectively. The respective percentages of up- and down-regulated genes generally ranged between 40 and 60% although some exceptions were noticed: thus the percentages of down-regulated genes reached around 75% for donor #3 treated with the low concentrations of ROSI and MURA. Much less variations in the number of deregulated genes, not exceeding 2-fold, were observed between the three passages of HepaRG cells. The percentages of down-regulated genes reached around 60% of total deregulated genes with low concentrations of ROSI and MURA and 300 µM TESA, whereas they represented only around 30% with 5 µM TRO.

**Table 1:** Up- and down-regulated genes by PPAR agonists in primary human hepatocytes and HepaRG cells.

Compound (µM)	Primary human hepatocytes				HepaRG cells			
	Donor	Total genes	Up-regulated	Down-regulated	Passage	Total genes	Up-regulated	Down-regulated
TRO(5)	1	2174	1295	879	9	182	116	66
	2	2910	1682	1228	10	144	94	50
	3	428	234	194	11	197	130	67
	4	872	315	557				
	combined data	174	111	63	combined data	152	109	43
TRO(20)	1	4281	2274	2007	9	335	208	127
	2	2763	1518	1245	10	328	160	168
	3	835	649	186	11	560	308	252
	4	1180	451	729				
	combined data	276	158	118	combined data	391	230	161
TRO(40)	3	1640	1113	527	9	1242	635	607
	4	1142	453	689	10	1579	883	696
					11	1056	546	510
	combined data	458	220	238	combined data	1314	733	581
ROSI(50)	1	716	327	389	9	1169	545	624
	2	3135	1396	1739	10	1282	607	675
	3	1160	419	741	11	781	355	426
	4	747	349	398				
	combined data	240	121	119	combined data	938	453	485
ROSI(100)	1	870	349	521	9	3240	1473	1767
	2	4408	2014	2394	10	3761	1730	2031
	3	2165	786	1379	11	2128	953	1175
	4	2104	863	1241				
	combined data	303	124	179	combined data	2868	1295	1573
ROSI(150)	1	4215	2196	2019	9	7104	3234	3870
	2	5527	2546	2981	10	6954	3162	3792
	3	2871	1416	1455	11	6619	3264	3355
	4	5274	2485	2789				
	combined data	2735	1390	1345	combined data	6855	3166	3689
MURA(50)	1	1950	886	1064	9	1134	448	686
	2	1642	858	784	10	1040	550	490
	3	2678	867	1811	11	825	371	454
	4	3089	1171	1918				
	combined data	597	240	240	combined data	744	328	416
MURA(100)	1	4061	1845	2216	9	2924	1221	1703
	2	2405	1002	1403	10	2880	1180	1700
	3	4224	1683	2541	11	1952	641	1311
	4	4490	1702	2788				
	combined data	1473	575	898	combined data	2363	903	1460
MURA(150)	1	7834	3832	4002	9	7662	3628	4034
	2	4933	2156	2777	10	7189	2996	4193
	3	8194	4032	4162	11	7541	3516	4025
	4	8637	4134	4503				
	combined data	5004	2489	2515	combined data	7552	3510	4042
TESA(300)	1	2906	1177	1729	9	858	408	450
	2	2111	980	1131	10	902	335	567
	3	3277	1294	1983	11	874	429	445
	4	2479	862	1617				
	combined data	570	223	347	combined data	839	395	444

Genes were taken as differentially expressed when at least 1.5-fold change with p≤0.01.

Using the Rosetta resolver® software, data from each donor were combined to be representative of a virtual pool of the 4 hepatocyte populations treated with the different PPAR agonists. In such a situation the total numbers of deregulated genes, as well as the numbers of up- and down-regulated genes, were greatly reduced, although a concentration-related effect was still observed whatever the tested compound. The total number of modulated genes did not exceed 1000 genes in the combined human hepatocyte donors, except with 150 µM ROSI (2735 genes) and 100 and 150 µM MURA (approximately between 1500 and 5000 genes). The combined values of the three HepaRG cells passages were usually greater than 1000 genes with 40 µM TRO, and 100 and 150 µM ROSI and MURA.

### Hierarchical clustering

Clustering using both the Euclidian and the Pearson distances associated to the ward's min variance link heuristic criteria were used on combined data. The first dendrogram showed the unspecific toxic signatures with the highest concentration of MURA and ROSI in both cell models, while the second clustering allowed a clear separation between glitazones and glitazars in human hepatocytes. The same conclusions were obtained with HepaRG cells except with 20 µM MURA which was closed to 5 and 20 µM of TRO, likely because the low number of modulated genes (data not shown).

**Figure 6:**
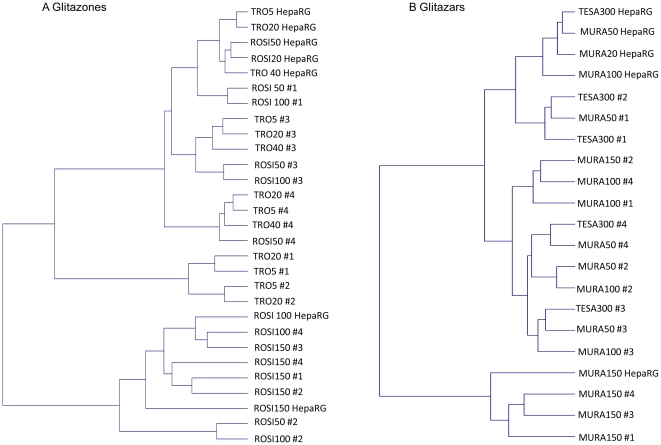
Two-dimensional hierarchical clustering of gene expression profiles in hepatocytes treated with PPAR agonists. The clustering was generated by using Resolver system software with an agglomerative algorithm Ward's min variance link heuristic criteria and Euclidean distance metric (FC≥1.5 and p≤0.01). Two-dimensional clustering was performed on gene expression profiles in hepatocytes treated with glitazones (a) and glitazars (b).

Two-dimensional hierarchical clustering of gene expression from human hepatocytes and HepaRG cells treated with either glitazones or glitazars was also performed (Figure 6). Two main clusters were demonstrated with both glitazones and glitazars. One branch grouped all hepatocyte 150 µM ROSI treatments and 50 or 100 µM ROSI treatments from donor #2. The second branch grouped all other treatments and was divided into 4 subtrees. Naturally, glitazone treatments were clustered more closely by donors than by treatment. After TRO treatment, donors #3 and #4 were closer to HepaRG cells than donors #1 and #2.

With glitazars, one of the two main branches grouped all 150 µM MURA treatments, except for donor #2. The other branch diverged into two distinct subtrees: one grouped all other HepaRG cell treatments and hepatocytes from donor #1 treated with 50 µM MURA and hepatocytes from donors #1 and #2 treated with TESA indicating that donors #1 and #2 were more similar to HepaRG cells than donors #3 and #4 under these treatment conditions.

### Functional analysis

Ingenuity pathway analysis was conducted on whole gene expression profiles in order to characterize the canonical pathways and biological and toxicological functions responsive to the four compounds at the different concentrations tested. Whatever the treatment and the cell model, these included “Fatty Acid (FA) metabolism”, “LPS/IL1 mediated inhibition of RXR function” and “Metabolism of xenobiotics”. In addition “Hepatic cholestasis” was identified with TRO treatment and “Bile acid metabolism” with the other three PPAR agonists. Finally, “PXR/RXR activation” was also identified with the two glitazones and “LXR/RXR activation” with the two glitazars.

### Gene expression analysis

The numbers of commonly modulated genes in the 4 human hepatocyte donors varied from 9 to 71% depending on PPAR agonist and concentration (Table 2). With respect to the total number of deregulated genes, these percentages ranged from 9 to 62%, 17 to 71%, 27 to 51°% and 31 to 48% with 5 µM TRO, 50 µM ROSI, 50 µM MURA and 300 µM TESA, respectively. By comparison, in the 3 HepaRG cell passages, these percentages ranged from 39 to 53%, 41 to 70%, 44 to 61% and 52 and 55% with 5 µM TRO, 50 µM ROSI, 50 µM MURA and 300 µM TESA, respectively. Accordingly, the calculated mean correlation coefficients were much lower between the hepatocyte donors than between the 3 HepaRG cell passages (Table 2). The percentages of commonly deregulated genes and the mean correlation coefficients increased with compound concentrations. Interestingly, these percentages were as low as 0.3, 2.5, 2.5 and 2.5% in the 4 human hepatocytes and 13.4, 24.4, 17.6 and 16.3% in the 3 HepaRG cell passages treated with 5 µM TRO, 50 µM ROSI, 50 µM MURA and 300 µM TESA, respectively (Table 3).

**Table 2:** Percentages of common modulated genes and correlation coefficients between donors in primary human hepatocytes and passages in HepaRG cells.

Compound	Concentration (µM)	Primary human hepatocytes					HepaRG cells			
		common modulatedgenes (%)				Mean correlation coefficients between donors	common modulated genes (%)			Mean correlation coefficients between passages
		donor number					passage number			
		1	2	3	4		9	10	11	
TRO	5	46.0	61.6	9.1	18.5	**0.25**	48.9	38.7	53.0	**0.69**
	20	68.2	44.0	13.3	18.8	**0.27**	40.7	39.8	68.0	**0.68**
ROSI	50	16.3	71.4	26.4	17.0	**0.47**	61.1	70.0	40.9	**0.75**
	100	12.6	63.8	31.3	30.4	**0.47**	67.2	78.1	44.2	**0.82**
	150	45.2	59.2	30.2	56.5	**0.64**	73.7	72.1	68.6	**0.82**
MURA	50	32.1	27.0	44.1	50.9	**0.38**	60.9	55.8	44.3	**0.66**
	100	47.9	28.4	49.9	53.0	**0.56**	71.3	70.2	47.6	**0.78**
	150	58.8	37.0	61.5	64.8	**0.62**	79.8	74.8	78.5	**0.89**
TESA	300	42.5	30.9	48.0	36.3	**0.26**	52.1	54.8	53.1	**0.62**

Percentages of common genes modulated in human hepatocytes from each donor or each HepaRG cells passage and correlation coefficients **(**FC≥1.5 and p≤0.01).

**Table 3:** Percentages of responsive genes modulated by PPAR agonists in primary human hepatocytes and in HepaRG cells.

Compound	Concentration (µM)	Common modulated genes (%)						
		Primary human hepatocytes				HepaRG cells		
		Number of donors				Number of passages		
		1	2	3	4	1	2	3
TRO	5	100.0	30.4	4.2	0.3	100.0	27.1	13.4
	20	100.0	37.6	5.9	0.8	100.0	33.4	14.7
	40					100.0	56.2	31.3
ROSI	50	100.0	21.2	7.2	2.5	100.0	44.4	24.4
	100	100.0	39.8	16.4	5.4	100.0	57.4	32.0
	150	100.0	52.4	27.7	11.4	100.0	68.4	45.9
MURA	50	100.0	33.8	9.5	2.5	100.0	36.5	17.6
	100	100.0	45.9	20.3	6.0	100.0	53.9	30.1
	150	100.0	64.8	37.2	14.3	100.0	75.8	58.7
TESA	300	100.0	37.7	9.2	2.5	100.0	36.2	16.3

Percentages of responsive genes modulated by PPAR agonists in primary human hepatocytes from one to four donors and in one to three passages of HepaRG cells.

**Figure 7:**
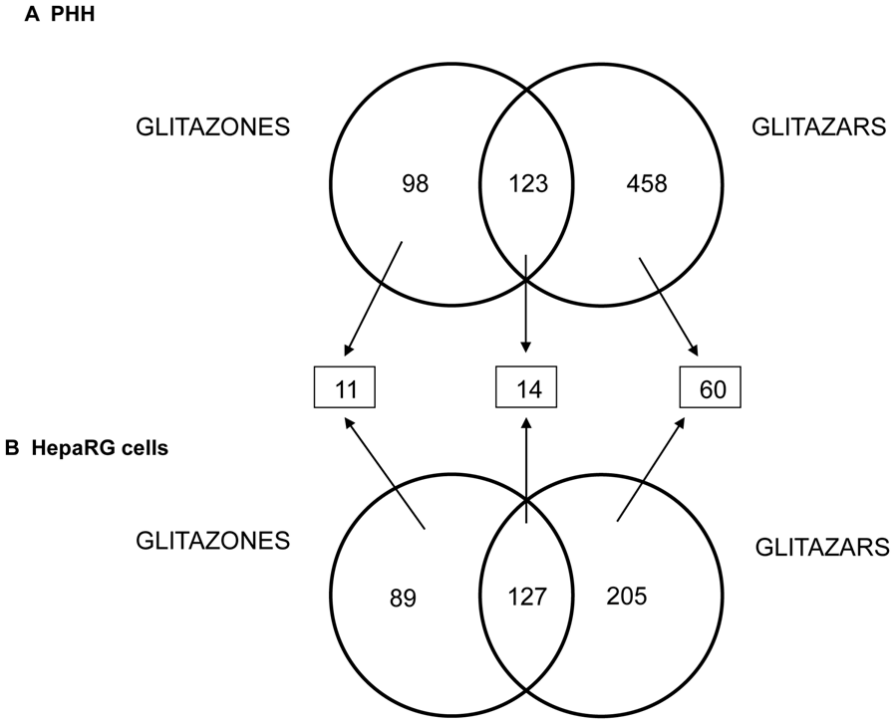
Venn diagram representation of differentially expressed genes in glitazone- and glitazar-treated hepatocytes. Venn diagrams showed overlap of gene signatures (FC≥1.5 and p≤0.01) in at least two concentrations of each glitazone and glitazar in PHH (a) and HepaRG cells (b).

Venn diagrams were performed on the differentially expressed genes in the PHH and HepaRG cells treated with the four agonists at different concentrations (Figure 7). In PHH, 123 deregulated genes were identified in PPARγ and PPARα/γ agonist treatments, while 98 and 458 deregulated genes were restricted to glitazones and glitazars, respectively. In the different HepaRG cell passages, 127 genes were common between PPARγ and PPARα/γ agonists, and 89 and 205 were restricted to glitazones and glitazars, respectively. By contrast, in both cell models, only a small set of 14 genes was identified as being deregulated in common by both glitazones and glitazars, while only 11 and 60 genes were deregulated in common by glitazones and glitazars, respectively. The 14 commonly altered genes by all PPAR agonists in both cell models included genes implicated in lipid metabolism (CD36, PLIN4, ADFP, ANGPTL4) and oxidative stress (POR, HMOX1). The 11 genes modulated only by the two glitazones in both culture models were involved in two main functions, namely, xenobiotic metabolism (CYP3A4, CYP3A7, ADH1B*)* and the immune system (CD14). As anticipated among the 60 genes deregulated only by glitazars, many were known as PPARα target genes (ACSL1, ACSL5, CPT1A, FGF21); other modulated genes were involved in calcium homeostasis (CD52, CC7) and cell-cell signalling (KCND2, CCR1, CXCL13).

However, many more deregulated genes were identified when only one cell model, an individual human hepatocyte population, a class of agonists or an individual agonist was specifically considered. However, whatever the condition, a majority of the altered genes were involved in various aspects of lipid metabolism, as well as other functions, including glucose, cholesterol and bile acids and amino acid metabolisms, chemical biotransformation, inflammation or immunity (Table 4). Several genes were specifically altered by glitazone treatment in one liver cell model only. Thus, in PHH, glitazone treatment affected expression of some genes involved in either xenobiotic metabolism (CYP2C8) or bile acid transport (SLCO1B3 also called OATP-8), while other genes were deregulated by only one of the 2 glitazones tested, (i.e. UCP2 by TRO and ALDH3A1, CYP1A2 and SLC10A2 by ROSI). Among the modulated genes restricted to HepaRG cells, AKR1B1 and AKR1B10 were deregulated by both glitazones, while ABCD3 and PCK1 were altered by TRO only and GPD2, HADHA, AKR1C3 and PC by ROSI only.

**Table 4:** Main target genes regulated by PPAR agonists in primary human hepatocytes and HepaRG cells.

	TRO		ROSI		MURA		TESA	
Lipid and hormone transport	**CD36**	SLC27A4	**CD36**	**SLC27A4**	**CD36**	**SLC27A4**	**CD36**	**SLC27A2**
				**SLC27A2**				
AcylcoA formation hydrolysis and binding	*ACSL5*	**FABP4**	**ACSL1**	FABP1	**ACSL1**	**FABP1**	**ACSL1**	*ACOT12*
	FABP1	*FABP5*	*ACSL3*	**FABP3**	**ACSL3**	**FABP3**	*ACSL3*	**FABP1**
	*FABP3*		**ACSL5**	**FABP4**	**ACSL5**	**FABP4**	*ACSL4*	**FABP3**
				*FABP5*		*FABP5*	**ACSL5**	*FABP4*
							*ACOT1*	*FABP5*
Mitochondrial β-oxidation and oxidative phosphorylation	CPT1A	SLC25A20	*ACADL*	**HADHA**	ACAA2	ETFDH	**ACAA2**	**ETFDH**
	CPT2	*TXNIP*	**CPT1A**	HADHB	*ACADL*	**HADHA**	**ACADM**	**HADHA**
	HADHA	UCP2	CPT2	SLC25A20	ACADVL	**HADHB**	*ACADS*	**HADHB**
			ETFDH	*TXNIP*	**CPT1A**	SLC25A20	**ACADVL**	**SLC25A20**
					CPT2	**TXNIP**	**CPT1A**	**TXNIP**
					CRAT	*UCP2/* UCP2	**CPT2**	
Ketogenesis and ketolysis	FGF21	HMGCS2	**BDH1**	HMGCS2	**BDH1**	HMGCS2	**FGF21**	**HMGCS2**
			**FGF21**		**FGF21**			
Peroxisomal β-oxidation	*ABCD3*	ECH1	*ABCD2*		*ABCD2*	*HACL1*	**ABCD3**	*HSD17B4*
	CROT	*HACL1*	*CROT*		**ABCD3**	HSD17B4	ACOX1	**PEX11A**
		*PEX11A*	ECH1	**PEX11A**	*CROT*	**PEX11A**	*ECH1*	
Microsomal ω hydroxylation	CYP4A11		*CYP4A11*	ALDH3A1	ALDH3A1	*CYP4A11*	**CYP4A11**	**CYP4X1**
			CYP4A11		CYP4A11	*CYP4X1*		
Lipogenesis	AGPAT6	ELOVL6	*AGPAT5*	*SCD*	*AGPAT5*	*SCD*		*ACACB*
			*AGPAT6*	*SLC25A10*	ELOVL6	SLC25A10		*AGPAT2*
			*HSD17B2*	SREBF1	FADS1			**MLYCD**
Lipase and lipid droplets	**ADFP**	**PLIN1**	**ADFP**	**PLIN1**	**ADFP**	*LIPE*	**ADFP**	*LIPE*
	CES1	**PLIN4**	**CIDEC**	**PLIN4**	CES1	**PLIN1**	*CES1*	**PLIN1**
	*CIDEC*		*LIPE*		**CIDEC**	**PLIN4**	*CES3*	**PLIN4**
	*LIPE*				*G0S2*	**PNPLA2**	**CIDEC**	
Lipoprotein uptake and metabolism	**ANGPTL4**		**ANGPTL4**	*LIPC*	*ANGPTL3*	*LIPC*	**ANGPTL4**	**APOA5**
	*APOA2*		*ANGPTL3*	*LPL/* LPL	**ANGPTL4**	*LPL*	APOA1	*LPL*
	*VLDLR*		*APOA1*	*MTTP*	**APOA1**	**MTTP**	*APOA2*	**VLDLR**
			*APOA2*	PLTP	*APOA2*	PLTP		
			*APOC3*	**VLDLR**	APOA5	VLDLR		
					*APOC3*			
Biotransformation	**ADH1B**	CYP2C8	**ADH1B**	*CYP2C9*	**ADH1B**	*CYP3A4*	**ADH1B**	*CYP2J2*
	*AKR1B1*	**CYP3A4**	**AKR1B1**	*CYP2E1*	**CYP1A1**	*CYP3A7*	**CYP1A1**	EPHX2
	*AKR1B10*	**CYP3A7**	**AKR1B10**	*CYP2J2*	CYP1A2	**EPHX2**	*CYP3A4*	*GSTA3*
	CYP2B6	*MGST3*	*AKR1C3*	**CYP3A4**	*CYP2C8*	**GSTA3**	*CYP3A7*	*MGST3*
			**CYP1A1**	**CYP3A7**	*CYP2C9*	*MGST3*		
			CYP1A2	**EPHX2**	*CYP2E1*			
			**CYP2B6**	*GSTA3*				
			CYP2C8	*MGST3*				
Amino acid metabolism		*OTC*	**ABAT**	*GPT*	**ABAT**	**GLS2**	**ABAT**	**CTH**
		**TAT**	*ACMSD*	**HAL**	*ACSMD*	*GPT*	*AGXT2*	**GLS2**
			**AGXT2**	**HPD**	**AGXT2**	*HAL*	*ARG1*	*OTC*
			*ARG1*	**OTC**	**ARG1**	**HPD**	**CBS**	*PAH*
			*CBS*	*PAH*	**CBS**	**OTC**		**TAT**
			**CTH**	**TAT**	**CTH**	*PAH*		
			**GLS2**		GLS	PSAT1		
						**TAT**		
Inflammation	*CCL2*	*CXCL10*	*APCS*	*MT1A*	APCS	*IL1B*	*BIRC3*	*IL1B*
	CCL3	*IL1B*	*CCL2*	*ORM2*	*BIRC3*	IL1RAP	*CRP*	**PLA1A**
	*CRP*	**VNN1**	CCL3	**PLA1A**	**CCL2**	*IL8*	CCL3	*VNN1/* VNN1
			*CD68*	**SAA4**	CCL3	*MT1A*	EMR1	
			*CRP*	*TRAF1*	CEBPB	*ORM2*		
			**CXCL10**	VCAM1	*CRP*	**PLA1A**		
			EMR1	*VNN1*	**CXCL10**	**SAA4**		
			**IL1B**					
Cholesterol/Bile tansport and metabolism	ABCB4	*SLC10A1*	**ABCB4**	**CYP7A1**	ABCB11	**CYP7A1**	**ABCB4**	CYP27A1
	**CYP7A1**	SLCO1B3	**ABCC2**	*CYP8B1*	**ABCB4**	**CYP8B1**	ABCB11	**SLC10A1**
			*ABCG5*	*NPC1*	**ABCG5**	EMR1	CAV1	*SLC10A2*
			*ABCG8*	**SLC10A1**	**ABCG8**	*NPC1*	**CYP7A1**	
			**CAV1**	SLC10A2	CAV1	**SLC10A1**		
			*CYP27A1*	SLCO1B3	**CYP27A1**	**VCAM1**		
Oxidative stress	**HMOX1**	**POR**	**HMOX1**	**POR**	**HMOX1**	**POR**	**HMOX1**	**POR**
		**OASL**	**OASL**			**OASL**		**OASL**
Immune system	*LECT2*	**MBL2**	*LECT2*	**MBL2**	**LECT2**	**MBL2**	*LECT2*	**MBL2**
Miscellanous	**CD14**	*KCND2*	**CD14**	*KCND2*	*CD14*	**KCND2**	*CD14*	**KCND2**
	*IRF7*	**SGK2**	*CD52*	**SGK2**	**CD52**	**SGK2**	**CD52**	**SGK2**
			**IRF7**		**IRF7**		**IRF7**	

Up-regulated genes: in both cell models (capital bold), primary human hepatocytes only (capital) and HepaRG cells only (italic). Down-regulated genes are underlined.

In PHH, glitazars caused deregulation of several genes involved in either lipid metabolism (ACADVL and ACAA2) or glucose homeostasis (GK). In addition, FADS1 and ACOX1, were induced by MURA and TESA respectively. In HepaRG cells, while PCK1 was modulated by both glitazars, ACADL and PNPLA2 deregulation was restricted to MURA and ACADS to TESA. Finally, some genes were found up-regulated by all PPAR agonists only in either PHH (HMGCS2, CCL3) or HepaRG cells (LIPE, IL1β, MGST3, CRP).

Additionally, both qualitative and quantitative differences were observed with several genes in response to the four compounds in the two cell models. Large variations in their extent of modulation were also observed with several genes in response to the four compounds. Thus, in HepaRG cells, changes in ADFP, FABP4 or ANGPTL4 expression induced by TRO, ROSI and MURA at equimolar concentrations (i.e. 20 µM) ranged between 2- and 5-fold. Similarly, huge interdonor variations were demonstrated in many responsive genes and several target genes were deregulated only in some donors. Thus, induction of FABP4 varied 3-fold and 13-fold among the donors treated with 20 µM TRO and 50 µM MURA, respectively. Similar variations were observed with some down-regulated genes. As an example, an 8-fold difference was observed in CYP7A1 down-regulation between the 4 donors after a 20 µM TRO treatment (Tables S3 and S4). Opposite regulation was also observed with a few genes between the donors, as, for example, for PDK4, which was up-regulated in donors #1 and #2 but down-regulated in donors #3 and #4 after treatment with 5 µM and 20 µM TRO. These results were confirmed by qPCR analysis (data not shown).

### Comparative microarray and qPCR data

Microarray and qPCR results were compared for several genes at the lowest concentration of each PPAR agonist and, in addition, at the middle concentration of TRO in HepaRG cells (Table 5). For each analysis the direction of change obtained by q-PCR was similar to that observed with microarrays and, as usually seen, qPCR values were often higher. As expected, transcripts encoding FABP4, PDK4, FABP1, ADFP and CYP3A4 were greater after treatment with the four PPAR agonists, while those of CYP2B6 increased only with the two glitazones. Expression of two genes representative of liver-specific functions, namely albumin and aldolase B, was not affected by any of the treatments, except for a slight increase in aldolase B with 300 µM TESA. PPARα expression was slightly induced by MURA while PPARγ expression was increased by 5 µM TRO and decreased by both MURA and TESA. Expression of HMOX-1, a gene encoding a protein involved in oxidative stress, was also slightly increased with 20 µM TRO.

**Table 5:** Validation by qPCR analysis of the microarray results in HepaRG cells exposed to PPAR agonists.

Gene name	qPCR results					Microarray results				
	TRO5 µM	TRO 20 µM	ROSI 20 µM	MURA 20 µM	TESA 300 µM	TRO 5 µM	TRO 20 µM	ROSI 20 µM	MURA 20 µM	TESA 300 µM
FABP4	12.9	24.1	45.8	41.2	73.9	6.5	8.0	2.7	13.5	15.7
PDK4	1.1	2.9	9.6	4.7	8.3	1.3	1.7	4.2	1.1	20.0
FABP1	2.2	2.6	2.1	6.1	4.5	1.7	1.4	1.3	2.0	2.8
ADFP	2.6	2.8	2.7	3.8	5.6	2.4	2.9	3.9	1.6	8.6
CYP3A4	2.5	4.1	16.5	3.9	7.9	2.5	3.3	4.4	-1.2	2.9
CYP2B6	1.5	1.9	3.7	-1.1	-1.0	1.3	1.1	1.3	-1.1	1.1
HMOX1	1.4	1.7	-1.1	-1.0	1.3	1.4	1.6	1.8	1.4	3.3
ALB	1.4	0.9	1.3	1.4	1.9	1.0	-1.4	-1.6	-1.0	-1.2
ALD-B	-1.3	0.6	-1.4	-1.4	-2.0	-1.2	-1.8	-1.9	-1.1	-2.1
PPARα	-1.3	0.7	1.4	1.8	-1.2	1.0	1.0	-1.3	1.1	-1.2
PPARγ1	1.9	1.3	-1.1	-1.7	-1.5	-1.1	-1.1	-1.1	1.0	-1.0

mRNA fold change for each gene corresponds to the ratio of mRNA expression in HepaRG cells treated by PPAR agonists for 24h *vs* their untreated counterparts; the values are means of at least two microarrays or three qPCR experiments.

### Activity of the NTCP transporter

**Figure 8:**
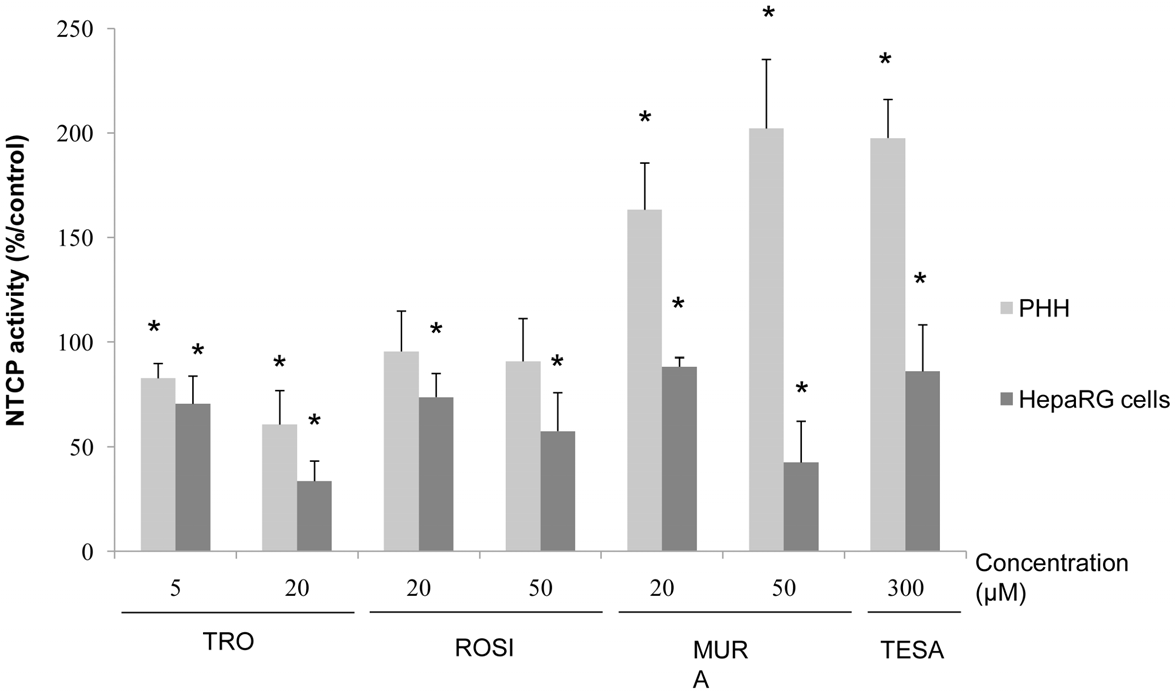
NTCP activity in primary human hepatocytes and HepaRG cells after 24 h treatment with PPAR agonists. NTCP activity was determined at two concentrations of TRO, ROSI, MURA or one concentration of TESA. Each bar chart colour represents a cell condition treatment. Results are normalized to control cells and expressed as means ± S.D.of three independent experiments.* p<0.05.

The activity of the NTCP transporter was estimated through measuring sodium-dependent intracellular accumulation of radiolabelled taurocholate substrate after a 30 min incubation period with 0.17 µM [^3^H] taurocholate in the presence or absence of sodium. In control cultures, NTCP activity in HepaRG cells represented around 50% of that measured in PHH. NTCP activity was much greatly reduced, decreasing to 70% vs 33% and to 8 2% vs 60% after treatment with 5 and 20 µM TRO, respectively (Figure 8). By contrast, the activity remained unchanged with ROSI and was enhanced 1.5 to 2.0-fold by MURA and TESA in PHH, while a decreased was noticed following ROSI and MURA treatment in HepaRG cells.

## Discussion

While the effects of PPARα activation on gene regulation in rodent and human liver have been extensively investigated [16]–[18], only a few studies have addressed hepatic gene regulation by PPARγ or PPARα/γ and have been restricted to the analysis of a small subset of genes [4]. To our knowledge, the present report describes the first analysis of overall gene expression profiles induced by PPARγ and PPARα/γ agonists in human hepatocytes, using the transcriptomic approach. Although our data highlighted huge variations in gene expression profiles depending on hepatocyte donor, the class of PPAR agonist, the specific agonist and its concentration, they also showed that PPARγ and PPARα/γ control a large number of common target genes and allowed the identification of subsets of genes regulated by only one PPAR isotype.

Previous studies have shown that the use of high chemical concentrations result in the deregulation of an increasing numbers of nonspecific genes, such as those involved in apoptosis/necrosis and cellular stress [19], thereby supporting the choice to analyse several chemical concentrations in our study. Our preliminary toxicity study using a molar comparison ranked TRO > ROSI ≈ MURA > TESA. In agreement with clinical and experimental studies, [20], [21] TRO was found to be the most cytotoxic compound, causing a significant decrease in intracellular ATP content at 40 µM, especially in HepaRG cells. The higher cytotoxicity associated with ROS production, of TRO compared to ROSI, in human hepatocytes has been previously reported [22] and found to be PPARγ-independent. This supports the view that TRO toxicity is a non-receptor mediated effect and is most likely the result of a primary interaction with mitochondria [23]. Accordingly, 40 µM TRO and 100 µM ROSI used in our study represented around 8- and 100-fold the therapeutic plasma concentration, respectively. This could explain the large safety margin observed with ROSI but not with TRO for hepatotoxicity.

To our knowledge, *in vitro* hepatotoxicity of glitazars had not been previously reported. In our study, while ATP loss and ROS production were observed from 100 µM MURA, no effect was observed with 300 µM and only a slight ATP decrease was noticed at 2000 µM TESA. One hundred µM MURA and 300 µM TESA correspond, respectively, to around a 70-fold and >400-fold therapeutic plasma concentration [24], which would agree with the absence of any liver damage reported in patients treated with these two compounds during clinical trials.

The gene expression profiles obtained within the 4 hepatocyte donors showed poor overlap. Indeed, similarity was only 0.3 to 14.4% of the total number of the genes deregulated in the 4 populations, whatever the test compound. Only a small subset of commonly deregulated genes was shared between hepatocytes from the 4 donors following treatment with low concentrations of the PPAR agonists. This is in agreement with the findings reported by Goyak et al., showing that the number of genes modulated in common in ten human hepatocyte donors treated with arochlor 1254, di(2-ethylhexyl)phthalate or phenobarbital, did not exceed 0.1% of the total deregulated genes [25].

Noteworthy, many genes that were induced only in several hepatocyte donors, were no longer modulated when the gene expression profiles of all 4 donors were combined. Thus, whereas FABP5 and PLIN4 were respectively induced at least 2.0- and 4.7-fold in two out of four hepatocyte donors after TESA treatment, their fold change was only 1.4 and 2.3 respectively when the four individual values were combined. Similarly, anti-correlated genes, such as PDK4, could not be detected after combining data.

Despite the large variable number of total deregulated genes and the low percentages of common deregulated genes between donors by the different compound treatments, some major metabolic pathways were found to be reproducibly modulated by both PPARγ and dual PPARα/γ agonists by Ingenuity Pathway Analysis®, especially fatty acid metabolism, LPS/IL1 mediated inhibition of RXR function and xenobiotic metabolism pathways. Accordingly, many specific genes targeting various lipid metabolic pathways were modulated by both PPARγ and PPARα/γ agonists, in at least several human hepatocyte donors and for some concentrations (Table S3). Thus, many genes involved in pharmacological PPAR targeted functions were up-regulated, including intracellular uptake and binding of fatty acids (SLC27A2, CD36, FABPs, PLIN, CIDEC, ADFP), hepatic ketogenesis (FGF21, HMGCS, BDH1), mitochondrial β-oxidation (ACAD, CPT2), peroxisomal β-oxidation (ABCD3, ECH1), lipogenesis (AGAPTs, SCD), lipolysis (PNPLA2, LIPE), lipoprotein metabolism (LPL, ANGPTL4) and glucose/glycerol metabolism (AQP7, GYS2, PDK4, TXNIP). The direction of changes induced by PPAR agonists was usually similar in both human hepatocytes and HepaRG cells. However, CYP4A11, the main human CYP of the CYP4A gene subfamily that catalyzes microsomal ω-hydroxylation of fatty acids was down-regulated in HepaRG cells while it was up-regulated in human hepatocytes. Also, ALDH3A1 was up-regulated by ROSI but down-regulated by MURA in human hepatocytes. Genes involved in other peroxisomal functions, such as amino acid (TAT, OTC) and cholesterol (CYP7A1, SLC10A1) metabolisms were negatively affected by all PPAR agonists tested. In addition, genes related to biotransformation (CYP3A4, MGST3), inflammation (SAA4, PLA1A), oxidative stress (HMOX1, POR) and immunity (MBL2, OASL) were found deregulated by both glitazones and glitazars.

Moreover, various genes previously described as specific PPARα target genes were found modulated not only by glitazars but also by glitazones. These genes were involved in numerous major biological functions such as lipoprotein metabolism (LIPC, PCTP), inflammation (VNN1), peroxisomal β-oxidation (PEX11A), and ketogenesis (FGF21). The fact that PPARα and PPARγ agonists can activate the same target genes is not surprising since both PPARα and PPARγ recognize similar DNA response elements. PPARα target genes have also been found to be deregulated by PPARγ agonists in liver of obese mice, without concomitant overexpression of PPARγ [26].

In addition, in the present study, several specific glitazar target genes were identified, these being involved in peroxisomal β-oxidation (ACAA2, ACADVL, ME1), lipoprotein metabolism (APOA5), lipogenesis (FAD) and inflammation (BIRC3) likely reflecting the involvement of the PPARα component of these molecules. By contrast, the up-regulation of the three xenobiotic metabolism genes AKRIB10, CYP2B6 and CYP2C8 was restricted to glitazones, in accordance with previous studies describing CYP2B6 and 3A4 induction by glitazones [27]–[29]. Noteworthy, it must be borne in mind that, in addition to limited qualitative differences in the gene sets deregulated by the two classes of agonists, major quantitative differences between ROSI and MURA at a 50 µM concentration were sometimes observed, as, for example, for PDK4, FGF21 and PLIN genes.

Moreover, many fewer genes were specifically modulated by TRO or TESA compared to ROSI or MURA in both PHH and HepaRG cells. These differences could be related to the higher affinity of these two latter compounds for PPARs. Indeed, in transfected CV-1 cells containing GAL4-PPAR chimeras, on the basis of EC_50_ values (i.e. concentrations which exhibited 50% efficacy) the affinity of MURA and TESA for PPARα was 0.32 and 9.44 µM, respectively, and that of TRO, ROSI, MURA and TESA for PPARγ was 2.24, 0.02, 0.11 or 1.82 µM, respectively [30]–[32]. Interestingly, cytotoxicity level and hierarchical clustering analysis also showed that ROSI was more similar to MURA than to TRO. These data are compatible with the recent withdrawal of ROSI from the market due to incidents of cardiotoxicity, as observed with MURA during clinical trials [33], [34].

Furthermore, some genes previously reported to be specifically deregulated in mouse cells, (e.g. CD36, UCP2, PNPLA2, LIPE [17]) were also found to be altered in human hepatocytes. Such discrepancies could be related to different experimental conditions or the hepatocyte donors. Other genes thought to be Kupffer cell markers, such as CD68 [35] and CD14 [36], were also found to be altered in human hepatocytes treated with either PPARγ or PPARα/γ agonists. LPL, described as a PPARα target gene restricted to Kupffer cells [37] was also always overexpressed, as well as its modulating factors such as APOC3 and ANGPTL4. Since these genes were also found to be responsive in HepaRG cells, this fact excluded the possibility of their induction by a few Kupffer cells contaminating the hepatocyte primary cultures.

Generally, in agreement with previous studies [9], [10], [19], [38], [39], the human hepatoma HepaRG cell line appeared to behave as a primary human hepatocyte population. Hierarchical clustering analysis showed that some human hepatocytes populations were closer to HepaRG cells than other human populations. However, some differences were observed. Thus, acute-phase genes were more deregulated in HepaRG cells than in human hepatocytes, (e.g. IL-1β and CRP).

Induction of cholestasis by TRO has already been commented upon [40]. In our study, we demonstrated that both glitazars and glitazones decreased the expression of both CYP7A1 and SLC10A1 (also called NTCP) which are involved in bile acid biosynthesis and transport, respectively. Moreover, down-regulation of NTCP has been described as an adaptative response to a decrease of ongoing intrahepatic cholestasis [41]. The down-regulation of NTCP expression observed in microarrays was confirmed at the activity level. NTCP activity was inhibited with TRO in human hepatocytes, supporting the cholestatic effect of this compound *in vivo*, as previously described [42]. Unexpectedly, following treatment with glitazars, NTCP activity was increased in human hepatocytes, while it was decreased in HepaRG cells. Further studies are required to explain such discrepancies.

In summary, this first global analysis of gene regulation by PPARγ and PPARα/γ agonists in human hepatocytes shows that despite major inter-individual variability, a large set of genes involved in various aspects of lipid metabolism and several other biological processes, was regulated in common leading to the conclusion that PPARα/γ and PPARγ agonists control a large number of common target genes. Only a small set of genes was specifically deregulated by glitazones. These data give new insights into the molecular mechanisms of action of PPARγ and PPARα/γ agonists and the large inter-individual and unpredictable response of patients to these compounds.

## Supporting Information

Table S1 Characteristics of the human liver donors.
(DOC)

Table S2 Sequences of primer pairs used.
(DOC)

Table S3 Genes involved and fold change after PPAR treatment in main metabolic pathways are listed in this table. The minimum and maximum fold changes obtained with primary human hepatocytes are indicated in square brackets.
(DOC)

Table S4 Genes involved and fold change after PPAR treatment in main metabolic pathways are listed in this table. The minimum and maximum fold changes obtained with HepaRG cells are indicated in square brackets.
(DOC)
