# Supplementary material for: Comparative Gene Expression Profiles Induced by PPARγ and PPARα/γ Agonists in Human Hepatocytes
Source: PLoS One. 2011 Apr 18;6(4):e18816. doi: 10.1371/journal.pone.0018816 (PMC3078935; doi:10.1371/journal.pone.0018816)
Supplement: Table S1 — Characteristics of the human liver donors. (DOC) [file pone.0018816.s001.doc]

**Table S1: Characteristics of the human liver donors**

| Donor | Sex | Age (years) | Pathology | Smoker |
| --- | --- | --- | --- | --- |
| 1 | Male | 48 | Metastasis | NA |
| 2 | Male | 75 | Metastasis | Yes |
| 3 | Male | 75 | Metastasis | Yes |
| 4 | Female | 81 | Metastasis | No |
