# Supplementary material for: Comparative Gene Expression Profiles Induced by PPARγ and PPARα/γ Agonists in Human Hepatocytes
Source: PLoS One. 2011 Apr 18;6(4):e18816. doi: 10.1371/journal.pone.0018816 (PMC3078935; doi:10.1371/journal.pone.0018816)
Supplement: Table S2 — Sequences of primer pairs used. (DOC) [file pone.0018816.s002.doc]

### Table S2 : Primer sequences used for qPCR analysis

| Genes | Forward primer | Reverse primer |
| --- | --- | --- |
| 18S | CGCCGCTAGAGGTGAAATTC | TTGGCAAATGCTTTCGCTC |
| FABP4 | TGGTGGTGGAATGCGTCAT | GGTCAACGTCCCTTGGCTTA |
| PDK4 | CTCGCGCTACAGCCCG | GCATTTTCTGAACCAAAGTCCAG |
| FABP1 | CACCCCCTTGATATCCTTCC | TTCTCCGGCAAGTACCAACT |
| ADFP | CTCATGGGTAGAGTGGAAAAGGAGCATTGG | TTGGATGTTGGACAGGAGGGTGTGGCACGT |
| CYP3A4 | CTTCATCCAATGGACTGCATAAAT | TCCCAAGTATAACACTCTACACAGACAA |
| CYP2B6 | TTCCTACTGCTTCCGTCTATCAAA | GTGCAGAATCCCACAGCTCA |
| HMOX1 | ACTTTCAGAAGGGCCAGGT | TTGTTGCGCTCAATCTCCT |
| ALB | TGCTTGAATGTGCTGATGACAGG | AAGGCAAGTCAGCAGGCATCTCATC |
| ALDOb | GCATCTGTCAGCAGAATGGA | TAGACAGCAGCCAGGACCTT |
| PPARα | CATTACGGAGTCCACGCGT | ACCAGCTTGAGTCGAATCGTT |
| PPARγ1 | GAGAGATCCACGGAGCTGAT | AGGCCATTTTGTCAAACGAG |
