# Supplementary material for: Comparative Gene Expression Profiles Induced by PPARγ and PPARα/γ Agonists in Human Hepatocytes
Source: PLoS One. 2011 Apr 18;6(4):e18816. doi: 10.1371/journal.pone.0018816 (PMC3078935; doi:10.1371/journal.pone.0018816)
Supplement: Table S3 — Genes involved and fold change after PPAR treatment in main metabolic pathways are listed in this table. The minimum and maximum fold changes obtained with primary human hepatocytes are indicated in square brackets. (DOC) [file pone.0018816.s003.doc]

**Table S3 : Comparative gene expression changes in primary human hepatocytes induced by a 24 h treatment with** PPAR agonists

|  |  |  | TRO | | | ROSI | | | MURA | | | TESA |
| --- | --- | --- | --- | --- | --- | --- | --- | --- | --- | --- | --- | --- |
|  |  |  | 5 µM | 20 µM | 40 µM | 50 µM | 100 µM | 150 µM | 50 µM | 100 µM | 150 µM | 300 µM |
| Lipid and hormone transport | **SLC27A2** | **solute carrier family 27 (fatty acid transporter), member 2** | **1.3[-1.1;1.7]** | **1.3[1.0;2.4]** | **1.2[1.3;1.4]** | **1.8[1.2;5.8]** | **1.8[1.1;5.9]** | **-1.3[-2.4;2.5]** | **2.0[1.3;3.7]** | **1.4[1.1;2.9]** | **-1.8[-6.6;1.6]** | **2.1[1.2;3.4]** |
|  | **SLC27A4** | **solute carrier family 27 (fatty acid transporter), member 4** | **1.2[-1.1;1.4]** | **1.6[-1.0;2.3]** | **1.5[1.2;1.7]** | **1.5[-1.0;1.9]** | **1.8[-1.0;2.7]** | **2.4[1.9;3.3]** | **1.3[1.0;1.9]** | **1.9[1.5;2.5]** | **2.3[1.6;2.8]** | **1.3[-1.4;2.0]** |
|  | **CD36** | **CD36 molecule (thrombospondin receptor)** | **1.8[1.6;3.3]** | **3.0[2.4;4.8]** | **2.6[2.3;4.5]** | **3.3[2.1;7.5]** | **2.9[1.7;7.5]** | **2.1[1.5;6.5]** | **5.3[3.3;9.3]** | **2.9[1.8;5.3]** | **1.3[-3.2;2.3]** | **5.8[4.0;8.5]** |
|  | SLC27A1 | solute carrier family 27 (fatty acid transporter), member 1 | -1.1[-1.1;1.0] | -1.1[-1.2;1.0] | -1.2[-1.1;1.4] | -1.1[-1.4;1.0] | -1.1[-1.3;1.1] | -1.1[-1.4;1.1] | -1.1[-1.4;-1.0] | -1.3[-1.6;-1.0] | -1.3[-2.0;1.0] | 1.1[-1.3;1.2] |
| AcylcoA formation hydrolysis and binding | ACOT1 | acyl-CoA thioesterase 1 | 1.0[-1.1;1.1] | 1.0[-1.1;1.2] | 1.1[-1.1;1.2] | 1.0[-1.2;1.2] | 1.1[-1.4;1.1] | -1.0[-1.6;1.2] | 1.2[-1.0;1.4] | -1.1[-1.4;1.3] | -1.4[-2.5;-1.1] | 1.4[1.2;1.6] |
|  | ACOT12 | acyl-CoA thioesterase 12 | 1.1[-1.3;1.4] | 1.0[-1.6;1.2] | 1.1[1.0;1.4] | -1.0[-1.1;1.1] | -1.2[-2.0;-1.1] | -4.0[-6.1;-1.8] | 1.1[-1.2;1.3] | -1.4[-3.1;-1.2] | -3.8[-6.6;-4.4] | 1.1[-1.1;1.2] |
|  | ACOT7 | acyl-CoA thioesterase 7 | 1.2[-1.1;1.9] | 1.2[-1.1;1.8] | -1.1[-1.2;-1.1] | -1.3[-1.5;-1.2] | -1.6[-2.1;-1.0] | -2.9[-4.1;-1.9] | -1.2[-2.0;1.3] | -1.6[-2.7;-1.2] | -2.8[-6.3;-1.8] | -1.1[-1.8;1.3] |
|  | **ACSL1** | **acyl-CoA synthetase long-chain family member 1** | **1.0[-1.5;1.7]** | **1.0[-1.9;1.7]** | **1.7[1.4;2.0]** | **1.7[1.4;2.7]** | **2.0[1.3;2.7]** | **1.6[1.2;2.8]** | **2.4[2.2;4.3]** | **2.5[1.9;3.7]** | **1.4[1.3;2.2]** | **2.3[2.2;3.6]** |
|  | **ACSL3** | **acyl-CoA synthetase long-chain family member 3** | **-1.0[-1.4;1.3]** | **-1.1[-1.7;1.3]** | **1.1[-1.1;1.3]** | **1.4[1.3;1.6]** | **1.4[1.0;1.5]** | **1.5[1.1;1.3]** | **1.6[1.3;2.2]** | **2.1[1.9;2.5]** | **2.0[-1.0;2.5]** | **1.4[1.2;1.8]** |
|  | ACSL4 | acyl-CoA synthetase long-chain family member 4 | -1.2[-1.6;1.1] | -1.2[-1.5;1.2] | -1.1[-1.0;1.1] | 1.1[-1.4;1.5] | -1.0[-1.3;1.7] | 1.5[1.0;1.7] | 1.0[1.0;1.7] | -1.1[-1.0;1.6] | -1.1[-1.3;1.8] | 1.0[-1.1;1.7] |
|  | **ACSL5** | **acyl-CoA synthetase long-chain family member 5** | **1.3[1.1;1.7]** | **1.2[-1.0;1.8]** | **1.4[1.6;1.6]** | **1.8[1.4;4.8]** | **2.3[2.0;7.1]** | **2.4[1.7;5.9]** | **1.8[1.7;3.0]** | **2.6[2.4;4.6]** | **2.6[1.5;5.6]** | **2.0[1.8;3.2]** |
|  | ACSM3 | acyl-CoA synthetase medium-chain family member 3 | 1.2[1.1;1.9] | 1.2[-1.3;1.9] | 1.6[1.5;2.1] | -1.0[-1.0;1.3] | -1.1[-1.5;1.1] | -1.9[-2.3;-1.4] | 1.5[1.2;2.5] | 1.0[-1.1;1.2] | -1.6[-3.0;1.4] | 1.3[-1.0;1.7] |
|  | ACSS2 | acyl-CoA synthetase short-chain family member 2 | -1.1[-1.2;1.1] | -1.1[-1.3;1.1] | 1.0[1.1;1.3] | -1.4[-1.8;-1.0] | -1.1[-2.0;1.5] | 1.2[-1.1;2.5] | 1.0[-1.2;1.3] | 1.0[-1.2;1.4] | -1.1[-2.0;1.5] | -1.2[-1.5;1.0] |
|  | **FABP1** | **fatty acid binding protein 1, liver** | **2.0[1.2;5.6]** | **2.4[1.9;5.3]** | **2.9[2.4;3.4]** | **1.8[1.7;4.0]** | **1.7[1.6;3.3]** | **-1.7[-2.9;1.3]** | **3.0[2.7;5.2]** | **2.0[1.5;3.1]** | **-1.2[-5.3;1.8]** | **2.8[2.5;4.6]** |
|  | **FABP3** | **fatty acid binding protein 3, muscle and heart** | **1.2[1.0;1.6]** | **1.2[1.0;1.9]** | **1.1[-1.9;1.5]** | **2.1[1.6;3.6]** | **4.9[2.1;13.0]** | **7.7[6.1;12.9]** | **2.7[1.6;6.3]** | **3.8[2.2;10.4]** | **3.9[-2.0;7.0]** | **3.6[1.2;9.2]** |
|  | **FABP4** | **fatty acid binding protein 4, adipocyte** | **2.1[-1.0;3.0]** | **2.6[-1.0;4.6]** | **1.9[1.0;2.2]** | **2.2[-1.0;2.6]** | **1.3[-1.2;2.0]** | **15.0[2.1;35.2]** | **8.2[-1.2;13.0]** | **2.0[-1.1;2.5]** | **6.8[-1.1;10.5]** | **1.1[-1.1;1.2]** |
|  | **FABP5** | **fatty acid binding protein 5** | **-1.1[-1.2;1.2]** | **1.0[-1.1;1.6]** | **-1.1[1.1;1.2]** | **-1.1[-2.6;1.4]** | **-1.0[-2.5;1.3]** | **-1.2[-2.7;1.3]** | **1.5[1.4;2.4]** | **1.5[1.4;2.5]** | **1.5[-1.4;3.6]** | **1.4[1.1;2.6]** |
| Mitochondrial  β-oxidation and oxidative phosphorylation | **ACAA2** | **acetyl-Coenzyme A acyltransferase 2** | **1.2[-1.2;1.4]** | **1.2[-1.2;1.6]** | **1.4[1.3;1.6]** | **1.4[1.4;1.8]** | **1.4[1.3;1.8]** | **1.1[1.2;1.6]** | **1.9[1.6;2.3]** | **1.5[-1.0;2.0]** | **1.1[-1.3;1.3]** | **2.0[1.3;3.1]** |
|  | ACAD10 | acyl-Coenzyme A dehydrogenase family, member 10 | 1.3[-1.1;1.7] | 1.3[1.1;1.8] | 1.2[1.1;1.4] | -1.0[-1.2;1.2] | -1.0[-1.4;1.2] | -1.4[-1.7;-1.2] | -1.4[-2.1;-1.1] | -1.8[-2.1;-1.4] | -2.4[-3.4;-2.0] | -1.3[-2.0;-1.0] |
|  | ACAD8 | acyl-Coenzyme A dehydrogenase family, member 8 | 1.2[1.1;1.4] | 1.2[1.1;1.4] | 1.2[1.3;1.3] | 1.0[1.1;1.2] | 1.1[1.1;1.1] | -1.3[-1.6;1.0] | 1.2[1.1;1.4] | 1.0[-1.1;1.2] | -1.4[-2.1;1.0] | 1.2[-1.0;1.3] |
|  | ACADL | acyl-Coenzyme A dehydrogenase, long chain | -1.0[-1.1;1.2] | -1.2[-1.7;1.0] | -1.2[-1.3;-1.1] | -1.2[-1.3;-1.1] | -1.4[-1.7;-1.4] | -2.4[-2.6;-1.7] | 1.0[-1.2;1.1] | -1.4[-1.7;-1.1] | -2.2[-2.9;-1.9] | -1.1[-1.2;1.1] |
|  | **ACADM** | **acyl-Coenzyme A dehydrogenase, C-4 to C-12 straight chain** | **1.0[-1.3;1.2]** | **1.0[-1.7;1.3]** | **1.0[-1.1;1.2]** | **1.3[1.2;2.0]** | **1.3[1.1;1.6]** | **-1.1[-1.1;1.1]** | **1.9[1.9;2.6]** | **1.5[1.4;2.2]** | **-1.1[-2.2;1.1]** | **1.7[1.7;2.1]** |
|  | ACADS | acyl-Coenzyme A dehydrogenase, C-2 to C-3 short chain | 1.4[-1.1;3.0] | 1.4[1.1;2.9] | 1.1[1.2;1.2] | -1.1[-1.2;1.1] | -1.1[-1.4;1.1] | -1.4[-1.4;1.0] | -1.0[-1.2;1.3] | -1.5[-2.0;-1.1] | -2.0[-2.8;-1.6] | -1.2[-1.6;1.1] |
|  | **ACADVL** | **acyl-Coenzyme A dehydrogenase, very long chain** | **1.2[-1.0;1.5]** | **1.4[1.1;1.6]** | **1.4[1.3;1.7]** | **1.3[1.3;1.7]** | **1.5[1.4;1.9]** | **1.4[1.3;1.7]** | **1.6[1.2;2.1]** | **1.6[1.3;2.0]** | **1.5[1.5;1.8]** | **1.6[1.3;2.2]** |
|  | ACOT2 | acyl-CoA thioesterase 2 | 1.1[-1.1;1.2] | 1.1[1.1;1.2] | 1.2[1.0;1.2] | -1.0[-1.3;1.2] | -1.1[-1.4;1.2] | -1.1[-1.6;1.1] | 1.2[-1.1;1.7] | -1.1[-1.3;1.2] | -1.7[-2.6;-1.4] | 1.3[-1.0;1.6] |
|  | ACOT9 | acyl-CoA thioesterase 9 | 1.2[-1.0;1.5] | 1.2[-1.0;1.4] | 1.0[-1.1;-1.0] | 1.0[-1.1;-1.0] | 1.0[-1.1;1.1] | 1.2[-1.0;1.4] | 1.1[1.0;1.1] | 1.3[1.1;1.5] | 1.6[1.4;2.2] | 1.1[-1.0;1.3] |
|  | CPT1B | carnitine palmitoyltransferase 1B (muscle) | 1.3[1.0;2.0] | 1.4[-1.1;2.4] | 1.2[1.1;1.2] | 1.1[-1.1;1.5] | 1.2[1.0;1.5] | 1.4[1.2;1.4] | 1.0[-1.5;1.2] | 1.2[-1.3;1.5] | 1.2[1.1;1.4] | 1.0[-1.6;1.4] |
|  | **CPT2** | **carnitine palmitoyltransferase II** | **1.6[1.1;2.4]** | **1.8[1.6;2.6]** | **2.2[2.2;2.6]** | **1.5[1.4;2.7]** | **1.7[1.3;2.3]** | **-1.3[-1.6;1.2]** | **2.1[1.9;2.8]** | **1.9[1.3;3.3]** | **-1.0[-1.9;2.1]** | **1.8[1.5;2.5]** |
|  | **CRAT** | **carnitine acetyltransferase** | **1.0[-1.5;1.4]** | **1.2[-1.0;1.9]** | **-1.0[-1.1;1.0]** | **-1.3[-1.6;1.0]** | **-1.3[-2.0;1.1]** | **-1.2[-1.2;-1.1]** | **-1.5[-2.3;1.2]** | **-1.8[-2.2;-1.3]** | **-2.0[-2.3;-1.5]** | **-1.3[-2.6;1.2]** |
|  | ETFB | electron-transfer-flavoprotein, beta polypeptide | 1.0[-1.0;1.1] | 1.1[-1.0;1.2] | 1.0[1.0;1.2] | 1.1[1.1;1.3] | 1.1[-1.0;1.3] | -1.0[-1.2;1.3] | 1.1[-1.2;1.3] | -1.1[-1.5;1.2] | -1.5[-2.2;-1.0] | 1.0[-1.2;1.3] |
|  | **ETFDH** | **electron-transferring-flavoprotein dehydrogenase** | **1.1[-1.1;1.6]** | **1.3[1.1;1.8]** | **1.5[1.5;1.8]** | **1.5[1.8;1.9]** | **1.5[1.5;1.9]** | **-1.1[-1.2;1.6]** | **2.1[1.9;3.0]** | **1.6[1.4;2.3]** | **1.5[1.4;1.9]** | **2.0[2.1;2.7]** |
|  | HADH | hydroxyacyl-Coenzyme A dehydrogenase | 1.1[1.0;1.3] | 1.1[1.0;1.2] | 1.2[1.1;1.2] | 1.1[-1.2;1.5] | 1.2[-1.2;1.7] | -1.2[-1.8;1.5] | 1.3[1.0;1.5] | 1.2[1.0;1.3] | -1.5[-4.5;-1.1] | 1.2[1.1;1.5] |
|  | **HADHA** | **hydroxyacyl-Coenzyme A dehydrogenase alpha subunit** | **1.4[1.0;2.0]** | **1.6[1.3;2.1]** | **1.9[1.7;2.1]** | **1.6[1.3;2.4]** | **1.7[1.4;2.3]** | **1.1[-1.0;1.5]** | **2.5[1.9;3.2]** | **2.4[1.8;3.1]** | **1.8[1.3;2.6]** | **2.0[1.5;2.9]** |
|  | **HADHB** | **hydroxyacyl-Coenzyme A dehydrogenase beta subunit** | **1.3[1.2;1.5]** | **1.4[1.3;1.6]** | **1.5[1.5;1.6]** | **1.6[1.5;2.0]** | **1.7[1.6;1.9]** | **1.2[1.3;1.5]** | **2.5[2.3;3.2]** | **2.2[2.1;2.9]** | **2.0[1.6;2.3]** | **2.3[2.3;2.7]** |
|  | SLC22A5 | solute carrier family 22 (organic cation/carnitine transporter), member 5 | 1.0[-1.3;1.2] | -1.0[-1.3;1.1] | -1.1[-1.3;-1.1] | 1.2[-1.0;1.1] | 1.1[-1.0;1.1] | -1.0[-1.3;1.4] | 1.3[1.1;1.7] | 1.5[1.2;1.5] | 1.7[1.5;1.8] | 1.3[1.1;1.9] |
|  | **SLC25A20** | **solute carrier family 25, member 20** | **1.2[-1.0;1.7]** | **1.4[1.0;1.8]** | **1.7[1.9;2.0]** | **1.7[1.9;2.2]** | **1.8[1.7;2.3]** | **-1.4[-2.5;1.6]** | **2.5[1.7;3.8]** | **2.0[1.7;2.6]** | **-1.4[-2.5;1.9]** | **2.2[2.0;2.8]** |
|  | **TXNIP** | **thioredoxin interacting protein** | **-1.1[-1.3;1.3]** | **-1.0[-1.0;1.4]** | **-1.1[1.1;1.3]** | **1.0[-1.3;2.3]** | **1.1[-1.7;2.8]** | **1.8[1.6;2.5]** | **2.8[1.8;8.2]** | **4.0[2.1;12.5]** | **3.5[3.0;11.4]** | **2.4[1.9;8.4]** |
|  | UCP2 | uncoupling protein 2 (mitochondrial, proton carrier) | 1.6[-1.2;2.0] | 2.2[1.0;2.4] | -1.0[1.3;1.3] | 1.1[-3.6;2.8] | -1.1[-1.8;1.7] | -1.8[-2.8;1.4] | 1.0[-1.9;1.0] | -1.8[-3.1;-1.0] | -2.4[-3.0;-1.0] | -1.0[-1.7;1.5] |
|  | **CPT1A** | **carnitine palmitoyltransferase 1A (liver)** | **1.6[1.3;2.4]** | **1.7[1.5;2.6]** | **2.2[2.0;2.6]** | **2.5[1.7;4.6]** | **2.8[2.0;5.4]** | **3.4[1.7;7.1]** | **4.3[3.5;5.9]** | **5.1[4.1;7.5]** | **4.1[2.7;9.2]** | **3.1[2.5;4.0]** |
| Ketogenesis and ketolysis | ACAT1 | acetyl-Coenzyme A acetyltransferase 1 (acetoacetyl Coenzyme A thiolase) | -1.1[-1.5;1.0] | -1.1[-1.6;1.1] | -1.1[-1.1;-1.1] | -1.0[-1.0;1.0] | -1.1[-1.2;-1.1] | -1.3[-1.4;-1.0] | 1.1[-1.2;1.3] | -1.2[-1.6;-1.2] | -1.6[-2.5;-1.2] | 1.1[-1.1;1.0] |
|  | **BDH1** | **3-hydroxybutyrate dehydrogenase, type 1** | **1.2[-1.1;1.8]** | **1.2[-1.0;1.9]** | **1.0[1.1;1.1]** | **-1.4[-1.5;-1.1]** | **-1.5[-2.6;-1.3]** | **-4.4[-10.5;-2.8]** | **-1.1[-1.8;1.3]** | **-2.0[-6.9;-1.4]** | **-6.7[-25.4;-7.7]** | **-1.2[-2.4;1.4]** |
|  | **FGF21** | **fibroblast growth factor 21** | **1.5[1.2;2.6]** | **1.9[1.5;3.5]** | **1.4[1.5;2.1]** | **1.4[1.5;2.1]** | **2.1[2.0;3.1]** | **10.0[8.7;24.1]** | **2.8[2.6;3.9]** | **2.9[2.3;8.6]** | **9.2[4.4;50.8]** | **2.2[1.8;5.7]** |
|  | HMGCL | 3-hydroxymethyl-3-methylglutaryl-Coenzyme A lyase | 1.1[-1.1;1.3] | 1.1[-1.1;1.3] | 1.1[1.0;1.1] | -1.1[-1.2;-1.0] | -1.1[-1.2;-1.1] | -1.3[-1.2;-1.1] | -1.1[-1.3;-1.1] | -1.5[-1.8;-1.4] | -1.9[-2.3;-1.8] | -1.2[-1.5;-1.2] |
|  | **HMGCS2** | **3-hydroxy-3-methylglutaryl-Coenzyme A synthase 2 (mitochondrial)** | **1.1[-1.5;5.1]** | **1.4[-1.0;6.7]** | **2.2[2.9;7.0]** | **1.5[1.8;7.3]** | **1.7[1.9;3.5]** | **-1.7[-3.2;2.5]** | **2.9[5.9;13.7]** | **1.8[2.3;4.8]** | **-1.9[-15.1;-1.3]** | **3.0[6.5;13.9]** |
| Peroxisomal  β-oxidation | ABCD2 | ATP-binding cassette, sub-family D (ALD), member 2 | 1.1[-1.1;1.3] | 1.0[-1.1;1.1] | 1.0[-1.2;1.1] | -1.0[-1.2;-1.0] | -1.0[-1.2;1.0] | 1.1[-1.0;1.4] | 1.0[-1.1;1.0 | 2.2[-1.1;4.5] | -1.0[-1.2;1.2] | 1.1[-1.2;1.3] |
|  | **ABCD3** | **ATP-binding cassette, sub-family D (ALD), member 3** | **-1.1[-1.3;1.2]** | **-1.2[-1.7;1.2]** | **-1.0[-1.0;1.2]** | **1.3[1.3;1.7]** | **1.2[-1.1;1.5]** | **-1.5[-2.1;1.0]** | **1.8[1.7;2.3]** | **1.5[1.5;1.8]** | **-1.4[-3.2;1.4]** | **1.7[1.6;2.4]** |
|  | ACAA1 | acetyl-Coenzyme A acyltransferase 1 | 1.2[1.0;1.3] | 1.2[1.0;1.4] | 1.1[-1.0;1.2] | 1.0[-1.1;1.5] | -1.1[-1.5;1.4] | -2.0[-2.6;-1.4] | 1.3[-1.3;2.2] | -1.2[-2.1;1.2] | -2.5[-6.9;-1.6] | 1.2[-1.3;1.8] |
|  | ACOT8 | acyl-CoA thioesterase 8 | 1.1[1.0;1.2] | 1.0[-1.1;1.2] | -1.0[-1.0;1.0] | -1.2[-1.3;-1.1] | -1.1[-1.3;1.0] | -1.4[-1.5;-1.1] | -1.1[-1.4;1.1] | -1.3[-1.8;-1.0] | -1.6[-2.8;-1.3] | -1.1[-1.5;1.2] |
|  | ACOX1 | acyl-Coenzyme A oxidase 1, palmitoyl | 1.3[1.0;1.4] | 1.3[1.1;1.6] | 1.4[1.2;1.3] | 1.4[1.2;1.5] | 1.4[1.2;1.5] | 1.2[1.1;1.2] | 1.5[1.4;1.7] | 1.3[1.1;1.9] | -1.1[-1.7;1.4] | 1.6[1.3;1.8] |
|  | **CROT** | **carnitine O-octanoyltransferase** | **-1.3[-2.0;-1.5]** | **-1.6[-2.4;-1.7]** | **-1.5[-2.4;-1.3]** | **-1.1[-1.6;1.0]** | **-1.3[-2.1;-1.2]** | **-2.2[-4.8;-1.8]** | **-1.1[-2.0;-1.2]** | **-1.4[-2.1;-1.6]** | **-2.3[-9.7;-2.5]** | **-1.0[-1.6;1.1]** |
|  | DECR1 | 2,4-dienoyl CoA reductase 1, mitochondrial | -1.0[-1.1;-1.0] | -1.1[-1.3;-1.0] | -1.1[-1.1;-1.1] | -1.1[-1.1;-1.0] | -1.1[-1.3;-1.1] | -1.5[-1.6;-1.2] | -1.0[-1.1;-1.0] | -1.2[-1.5;-1.1] | -1.7[-2.8;-1.5] | -1.0[-1.1;-1.0] |
|  | DECR2 | 2,4-dienoyl CoA reductase 2, peroxisomal | -1.0[-1.1;1.0] | -1.0[-1.1;1.0] | -1.0[-1.1;1.0] | -1.1[-1.1;-1.0] | -1.1[-1.2;1.0] | -1.2[-1.4;-1.1] | -1.2[-1.6;1.0] | -1.6[-2.6;-1.1] | -2.5[-5.6;-1.8] | -1.2[-1.6;1.0] |
|  | **ECH1** | **enoyl Coenzyme A hydratase 1, peroxisomal** | **1.4[1.2;1.8]** | **1.7[1.4;2.1]** | **1.6[1.4;1.7]** | **1.5[1.4;1.8]** | **1.6[1.5;2.1]** | **1.1[1.0;1.4]** | **1.4[1.2;2.0]** | **1.4[1.1;1.6]** | **-1.1[-2.0;1.2]** | **1.4[1.1;1.9]** |
|  | EHHADH | enoyl-Coenzyme A, hydratase | 1.0[-1.1;1.1] | -1.1[-1.9;1.1] | -1.0[-1.1;1.1] | 1.1[1.3;1.5] | 1.1[-1.2;1.2] | -1.9[-3.0;-1.1] | 1.4[1.3;1.8] | -1.1[-1.7;1.3] | -2.0[-5.8;-1.8] | 1.3[1.2;1.8] |
|  | HACL1 | 2-hydroxyacyl-CoA lyase 1 | -1.0[-1.1;1.1] | 1.0[-1.0;1.1] | 1.1[1.1;1.1] | 1.1[1.0;1.3] | -1.0[-1.4;1.3] | -1.4[-1.5;-1.1] | 1.2[1.0;1.5] | 1.1[-1.1;1.2] | -1.2[-1.6;-1.0] | 1.3[1.2;1.5] |
|  | HSD17B4 | hydroxysteroid (17-beta) dehydrogenase 4 | 1.0[-1.2;1.2] | -1.0[-1.5;1.3] | 1.2[1.3;1.3] | 1.3[1.3;1.7] | 1.3[1.1;1.5] | -1.0[-1.1;1.3] | 1.7[1.5;2.0] | 1.3[1.1;1.5] | -1.0[-1.6;1.5] | 1.4[1.3;1.6] |
|  | PECI | peroxisomal D3,D2-enoyl-CoA isomerase | -1.0[-1.2;1.2] | -1.1[-1.3;1.1] | -1.0[1.0;1.2] | -1.1[-1.1;1.1] | -1.2[-1.3;1.0] | -1.5[-1.6;-1.2] | -1.1[-1.2;1.1] | -1.3[-1.8;-1.1] | -2.1[-3.0;-1.7] | -1.1[-1.3;1.1] |
|  | **PEX11A** | **peroxisomal biogenesis factor 11A** | **1.3[1.2;1.7]** | **1.3[1.2;1.8]** | **1.5[1.6;2.0]** | **1.9[1.8;3.2]** | **2.3[2.0;3.7]** | **1.2[-1.1;1.9]** | **3.0[2.7;5.3]** | **3.2[3.2;3.9]** | **1.5[-1.1;3.9]** | **2.7[2.4;4.1]** |
| Microsomal  ω hydroxylation | **ALDH3A1** | **aldehyde dehydrogenase 3 family, memberA1** | **-1.1[-1.5;2.8]** | **-1.1[-1.0;2.8]** | **-1.4[-1.1;1.4]** | **1.4[-1.1;14.3]** | **1.8[1.0;16.0]** | **1.8[-1.1;11.1]** | **-1.4[-1.6;2.3]** | **-2.3[-4.2;2.6]** | **-1.7[-2.6;3.4]** | **-1.4[-2.3;3.6]** |
|  | ALDH3A2 | aldehyde dehydrogenase 3 family, member A2 | 1.1[-1.1;1.3] | -1.0[-1.2;1.1] | 1.0[-1.1;1.1] | 1.1[-1.2;1.6] | 1.1[-1.2;1.4] | -1.5[-2.2;1.0] | -1.1[-1.3;1.0] | -1.2[-1.5;-1.1] | -2.5[-5.9;-1.4] | -1.3[-1.7;-1.2] |
|  | ALDH9A1 | aldehyde dehydrogenase 9 family, member A1 | 1.0[-1.2;1.3] | 1.0[-1.2;1.3] | 1.1[-1.1;1.3] | -1.0[-1.1;1.2] | -1.2[-1.4;1.0] | -1.6[-1.8;-1.3] | 1.2[-1.0;1.3] | -1.0[-1.1;1.3] | -1.5[-2.0;1.1] | 1.0[-1.1;1.1] |
|  | **CYP4A11** | **cytochrome P450, family 4, subfamily A, polypeptide 11** | **1.2[1.1;2.0]** | **1.3[1.2;2.0]** | **1.7[1.6;1.9]** | **1.6[1.9;2.8]** | **1.3[-1.3;2.4]** | **-1.3[-1.6;1.0]** | **2.1[2.4;6.4]** | **1.1[-1.9;3.6]** | **-1.6[-3.7;-1.9]** | **2.6[2.6;6.9]** |
|  | **CYP4X1** | **cytochrome P450, family 4, subfamily X, polypeptide 1** | **-1.0[-1.5;1.8]** | **-1.0[-1.3;1.7]** | **1.1[1.3;1.7]** | **1.5[1.7;2.3]** | **1.2[-1.4;2.2]** | **-2.0[-2.5;1.0]** | **2.5[2.5;4.1]** | **-1.0[-1.7;1.8]** | **-2.3[-4.0;-1.7]** | **3.1[3.2;4.6]** |
| Lipogenesis | ACACA | acetyl-Coenzyme A carboxylase alpha | 1.1[1.0;1.1] | 1.2[1.1;1.2] | 1.3[-1.0;1.5] | 1.0[-1.3;1.1] | 1.0[-1.4;1.2] | -1.1[-1.4;1.1] | 1.2[1.1;1.4] | 1.3[1.1;1.5] | 1.1[-1.7;1.7] | 1.1[-1.1;1.3] |
|  | ACACB | acetyl-Coenzyme A carboxylase beta | 1.0[-1.2;1.3] | 1.1[-1.1;1.3] | 1.1[1.1;1.3] | -1.1[-1.1;1.1] | 1.0[-1.2;1.3] | -1.5[-2.1;1.2] | 1.0[-1.3;1.4] | -1.1[-1.8;1.5] | -1.6[-3.5;-1.3] | -1.2[-1.6;1.1] |
|  | **AGPAT2** | **1-acylglycerol-3-phosphate O-acyltransferase 2** | **1.3[-1.0;1.8]** | **1.4[1.0;2.1]** | **1.1[-1.2;1.7]** | **1.0[-1.2;1.6]** | **1.1[-1.4;1.7]** | **1.5[1.1;1.8]** | **-1.1[-1.9;2.0]** | **-1.1[-1.8;1.5]** | **-1.6[-2.4;1.1]** | **-1.0[-2.8;2.1]** |
|  | AGPAT5 | 1-acylglycerol-3-phosphate O-acyltransferase 5 | -1.1[-1.3;1.1] | -1.1[-1.1;1.2] | 1.0[-1.1;1.2] | 1.3[1.2;1.8] | 1.3[1.2;2.1] | 1.4[1.4;2.0] | 1.0[-1.1;1.2] | 1.3[-1.0;1.6] | 1.5[-1.1;2.5] | 1.1[-1.1;1.4] |
|  | **AGPAT6** | **1-acylglycerol-3-phosphate O-acyltransferase 6** | **1.6[-1.4;3.6]** | **1.7[-1.2;4.3]** | **1.1[1.0;1.3]** | **-1.1[-1.6;1.4]** | **1.2[-1.9;1.8]** | **1.1[-1.1;1.6]** | **-1.4[-2.3;1.4]** | **-1.4[-1.8;-1.1]** | **-2.0[-3.1;-1.3]** | **-1.4[-3.8;1.2]** |
|  | DGAT1 | diacylglycerol O-acyltransferase homolog 1 (mouse) | 1.1[-1.1;1.4] | 1.2[-1.2;1.7] | 1.2[1.1;1.3] | 1.0[-1.0;1.3] | 1.0[1.0;1.1] | -1.1[-1.2;-1.1] | -1.1[-1.2;1.1] | -1.2[-1.4;-1.0] | -1.5[-1.7;-1.2] | -1.1[-1.2;1.0] |
|  | **ELOVL6** | **ELOVL family member 6, elongation of long chain fatty acids** | **1.4[1.3;1.7]** | **1.5[1.3;1.8]** | **1.9[2.0;2.0]** | **1.3[1.3;1.6]** | **1.5[1.2;2.0]** | **1.2[-1.2;2.9]** | **1.4[1.1;1.9]** | **2.4[1.6;4.0]** | **2.1[1.3;4.3]** | **1.4[1.2;1.8]** |
|  | ELOVL7 | ELOVL family member 7, elongation of long chain fatty acids (yeast) | 1.4[-1.6;2.5] | -1.1[-2.2;1.2] | -1.2[-2.1;-1.5] | -1.3[-2.5;-1.0] | -1.3[-3.4;-1.2] | 1.2[-1.5;-1.3] | 1.3[-1.5;2.0] | 1.0[-1.5;1.8] | 3.3[1.4;4.9] | -1.4[-2.4;1.2] |
|  | **FADS1** | **fatty acid desaturase 1** | **1.1[1.0;1.3]** | **1.1[1.0;1.3]** | **1.1[1.0;1.3]** | **1.1[1.2;1.4]** | **1.5[1.4;2.0]** | **1.4[1.3;2.6]** | **1.1[-1.0;1.3]** | **1.9[1.1;3.0]** | **2.2[1.4;4.5]** | **1.1[1.0;1.3]** |
|  | FASN | fatty acid synthase | 1.1[-1.0;1.7] | 1.2[1.2;1.5] | 1.2[1.1;1.4] | -1.1[-1.1;1.2] | -1.0[-1.6;1.2] | -1.6[-4.5;1.3] | -1.1[-1.6;1.4] | -1.3[-1.6;-1.2] | -2.3[-4.4;-2.0] | -1.2[-2.2;1.2] |
|  | GPAM | glycerol-3-phosphate acyltransferase, mitochondrial | -1.1[-1.4;1.1] | -1.1[-1.7;1.1] | -1.1[-1.2;-1.0] | -1.1[-1.9;1.2] | -1.1[-2.7;1.3] | 1.1[1.1;1.2] | 1.0[-1.3;1.3] | 1.1[-1.6;1.4] | -1.1[-1.8;1.1] | -1.1[-1.6;1.2] |
|  | HSD17B2 | hydroxysteroid (17-beta) dehydrogenase 2 | 1.1[-1.1;1.3] | 1.1[-1.0;1.3] | 1.0[1.1;1.3] | 1.0[1.0;1.5] | 1.1[-1.1;1.3] | -1.5[-1.7;1.2] | -1.0[-1.2;1.1] | -1.3[-3.4;-1.0] | -2.0[-5.2;-1.7] | -1.0[-1.2;1.1] |
|  | LPIN2 | lipin 2 | -1.0[-1.1;1.2] | -1.1[-1.2;1.1] | -1.1[-1.2;1.2] | -1.3[-1.4;-1.1] | -1.5[-1.7;-1.2] | -1.1[-1.2;1.1] | -1.3[-1.5;1.0] | -1.4[-1.4;-1.1] | -1.2[-1.4;1.7] | -1.3[-1.6;1.0] |
|  | **MLYCD** | **malonyl-CoA decarboxylase** | **1.2[1.1;1.3]** | **1.3[1.2;1.4]** | **1.3[1.3;1.3]** | **1.2[1.3;1.6]** | **1.4[1.3;1.6]** | **-1.1[-1.3;1.2]** | **1.5[1.2;2.2]** | **1.4[1.3;1.6]** | **1.2[1.1;1.5]** | **1.5[1.1;1.7]** |
|  | MOGAT1 | monoacylglycerol O-acyltransferase 1 | -1.4[-2.6;1.2] | -1.3[-2.2;1.2] | 1.0[-1.1;1.3] | -1.0[1.1;1.4] | -1.0[-1.2;1.3] | -1.2[-1.3;1.0] | 1.0[-1.1;1.2] | -1.2[-1.9;1.1] | -1.8[-2.6;-1.7] | -1.4[-1.6;-1.1] |
|  | **SCD** | **stearoyl-CoA desaturase (delta-9-desaturase)** | **1.1[-1.1;1.4]** | **1.1[-1.1;1.5]** | **1.1[1.0;1.4]** | **-1.0[-1.4;1.5]** | **1.2[-1.4;1.8]** | **1.2[-1.6;1.9]** | **1.4[1.4;1.9]** | **1.7[1.7;2.5]** | **1.1[-3.4;1.9]** | **1.3[1.2;1.7]** |
|  | SLC25A10 | solute carrier family 25 member 10 | 1.0[-1.1;1.3] | 1.1[-1.2;1.4] | 1.0[-1.0;1.2] | -1.3[-1.4;-1.1] | -1.3[-1.6;1.1] | -1.7[-2.0;-1.2] | -1.4[-1.8;1.1] | -1.9[-2.5;-1.6] | -3.0[-3.7;-2.1] | -1.3[-2.0;1.1] |
|  | **SREBF1** | **sterol regulatory element binding transcription factor 1** | **1.0[-1.4;1.2]** | **-1.0[-1.3;1.2]** | **-1.0[-1.1;-1.1]** | **-1.3[-2.6;-1.0]** | **-1.5[-4.2;-1.1]** | **-2.0[-6.5;-1.4]** | **-1.2[-1.9;1.1]** | **-1.4[-2.0;-1.4]** | **-1.9[-3.8;-1.8]** | **-1.2[-1.7;-1.1]** |
| Lipase and lipid droplets | **ADFP** | **adipose differentiation-related protein** | **1.3[-1.0;2.2]** | **1.6[1.0;2.7]** | **2.0[2.5;2.7]** | **2.8[3.6;6.5]** | **5.2[3.5;9.6]** | **8.8[12.2;12.3]** | **4.9[4.3;8.3]** | **8.5[5.2;19.1]** | **16.0[9.8;47.3]** | **5.3[5.9;7.1]** |
|  | **CES1** | **carboxylesterase 1 (monocyte/macrophage serine esterase 1)** | **1.4[-1.1;2.6]** | **1.5[-1.1;3.1]** | **-1.2[-1.1;1.0]** | **-1.2[-1.3;1.5]** | **1.0[-1.2;1.6]** | **1.0[-1.1;1.3]** | **-1.4[-1.6;1.1]** | **-1.6[-1.9;-1.3]** | **-2.5[-3.4;-1.9]** | **-1.3[-2.1;1.1]** |
|  | CES3 | carboxylesterase 3 (brain) | 1.0[-1.6;1.4] | 1.0[-1.6;1.5] | -1.1[-1.5;1.1] | -1.3[-1.7;-1.1] | -1.2[-1.9;1.1] | -1.3[-2.0;-1.1] | -1.2[-2.0;1.3] | -1.5[-2.7;-1.3] | -2.0[-3.2;-1.5] | -1.1[-2.1;1.2] |
|  | CIDEA | cell death-inducing DFFA-like effector a | 1.5[-2.8;2.8] | 1.4[-2.0;4.2] | 1.4[1.0;1.2] | 1.4[-1.3;3.0] | 1.3[-1.4;2.6] | 2.1[-1.1;5.9] | 1.1[-1.9;3.1] | 1.3[-2.7;3.5] | -1.4[-3.6;1.4] | -2.0[-2.3;1.4] |
|  | **CIDEC** | **cell death-inducing DFFA-like effector c** | **1.3[1.2;1.6]** | **1.3[1.1;1.7]** | **1.2[1.0;1.9]** | **1.6[1.3;3.3]** | **1.7[1.4;3.0]** | **1.6[1.2;2.7]** | **1.5[1.2;2.5]** | **2.3[1.6;3.7]** | **2.2[1.1;4.7]** | **1.6[1.1;3.0]** |
|  | **G0S2** | **G0/G1switch 2** | **-1.4[-1.7;1.0]** | **-1.5[-1.9;-1.1]** | **-1.6[-1.6;-1.3]** | **-1.0[-1.6;1.6]** | **-1.0[-1.2;1.7]** | **2.2[1.7;6.5]** | **-1.1[-1.6;1.5]** | **1.1[-1.7;2.7]** | **1.4[-1.1;3.4]** | **-1.1[-1.7;1.6]** |
|  | LIPA | lipase A, lysosomal acid, cholesterol esterase (Wolman disease) | -1.1[-1.2;1.1] | -1.2[-1.4;1.1] | -1.1[-1.1;1.0] | 1.0[1.0;1.3] | 1.1[-1.1;1.3] | -1.3[-1.3;1.1] | 1.3[1.2;1.4] | 1.1[-1.0;1.3] | -1.6[-4.3;-1.1] | 1.3[1.0;1.5] |
|  | LIPE | lipase, hormone-sensitive | -1.2[-1.4;1.0] | -1.0[-1.4;1.2] | 1.0[-1.3;1.5] | 1.1[-1.2;1.5] | 1.1[-1.1;1.5] | -1.0[-1.1;1.3] | 1.0[-1.3;8.1] | 1.3[1.1;6.7] | -1.0[-1.6;1.4] | 1.2[-1.2;5.6] |
|  | LSDP5 | lipid storage droplet protein 5 | 1.2[1.0;1.4] | 1.1[1.0;1.5] | 1.2[1.2;1.2] | -1.2[-1.5;-1.0] | -1.2[-2.0;1.0] | -1.6[-3.3;-1.2] | -1.1[-1.3;1.2] | -1.2[-1.4;-1.2] | -2.0[-2.8;-2.3] | -1.2[-1.5;-1.2] |
|  | MGLL | monoglyceride lipase | 1.1[1.0;1.4] | 1.1[-1.0;1.3] | 1.0[-1.1;-1.0] | 1.0[-1.1;1.0] | -1.0[-1.2;1.1] | 1.0[-1.2;1.1] | -1.1[-1.4;1.1] | -1.0[-1.1;1.3] | 1.1[-1.5;2.0] | -1.1[-1.3;1.2] |
|  | **KIAA1881** | **KIAA1881** | **2.3[1.2;3.8]** | **2.4[1.2;4.2]** | **1.5[1.1;1.5]** | **1.8[-1.0;2.5]** | **2.0[-1.1;2.9]** | **1.8[1.4;1.9]** | **2.1[1.1;4.7]** | **2.4[1.4;4.0]** | **-1.0[-4.1;3.8]** | **2.3[-1.0;5.5]** |
|  | **PLIN** | **perilipin** | **2.0[-1.2;6.6]** | **2.0[-1.0;5.2]** | **3.7[2.1;5.2]** | **6.5[2.4;18.2]** | **5.5[3.8;12.1]** | **2.1[1.4;4.5]** | **11.5[9.6;23.4]** | **6.7[2.7;20.1]** | **3.0[-1.3;6.0]** | **15.0[4.2;31.9]** |
|  | **PNPLA2** | **patatin-like phospholipase domain containing 2** | **1.4[-1.0;1.6]** | **1.6[-1.1;2.0]** | **1.4[1.1;1.4]** | **1.4[-1.0;1.7]** | **1.4[-1.4;1.7]** | **1.6[-1.2;2.1]** | **1.3[-1.5;2.5]** | **1.6[-1.1;2.8]** | **1.6[-1.2;2.5]** | **1.4[-1.8;2.7]** |
| Lipoprotein uptake and metabolism | **ANGPTL4** | **angiopoietin-like 4** | **1.2[-1.3;2.1]** | **1.5[1.3;2.7]** | **2.4[1.6;2.9]** | **3.5[2.0;12.7]** | **4.2[2.4;21.8]** | **4.8[2.5;15.6]** | **3.3[2.6;6.8]** | **4.9[3.9;9.1]** | **3.0[1.1;12.7]** | **4.7[3.6;9.8]** |
|  | **APOA1** | **apolipoprotein A-I** | **-1.0[-1.2;1.2]** | **-1.0[-1.2;1.2]** | **-1.5[-1.4;-1.3]** | **-1.2[-1.3;1.4]** | **-1.1[-1.3;1.2]** | **1.1[1.0;1.5]** | **-1.4[-2.0;-1.2]** | **-1.7[-2.5;-1.4]** | **-3.0[-11.9;-1.9]** | **-1.6[-1.7;-1.6]** |
|  | **APOA2** | **apolipoprotein A-II** | **1.2[1.0;1.3]** | **1.2[1.0;1.4]** | **1.1[1.1;1.1]** | **1.3[1.2;1.5]** | **1.5[1.2;1.6]** | **1.4[1.2;1.8]** | **1.5[1.1;1.9]** | **1.2[-1.7;2.0]** | **-1.0[-2.2;1.6]** | **1.4[-1.1;1.8]** |
|  | **APOA5** | **apolipoprotein A-V** | **1.1[-1.5;2.5]** | **1.3[1.1;2.6]** | **1.3[1.7;2.9]** | **1.5[2.7;10.9]** | **2.0[3.0;11.4]** | **-1.2[-1.7;3.3]** | **1.5[1.9;4.6]** | **1.5[-1.0;2.8]** | **-1.6[-9.6;1.6]** | **1.7[1.7;3.1]** |
|  | APOC3 | apolipoprotein C-III | -1.0[-1.3;1.1] | -1.1[-1.4;1.0] | -1.0[1.0;1.1] | -1.1[-1.0;1.3] | -1.0[-1.6;1.2] | -1.3[-1.3;-1.0] | 1.1[-1.0;1.2] | -1.3[-2.2;1.0] | -2.0[-3.7;-1.8] | 1.0[-1.1;1.2] |
|  | LIPC | lipase, hepatic | 1.0[-1.3;1.3] | -1.0[-1.5;1.3] | -1.0[-1.3;1.2] | 1.2[-1.0;1.4] | -1.1[-1.7;1.1] | -1.9[-2.3;-1.5] | 1.4[-1.3;1.8] | -1.0[-2.1;1.2] | -1.6[-2.8;-1.3] | 1.3[-1.3;2.1] |
|  | LIPG | lipase, endothelial | -1.3[-2.1;1.1] | -1.5[-2.8;-1.1] | -1.1[-1.2;-1.1] | 1.1[-1.0;1.1] | -1.1[-2.1;1.3] | -1.3[-4.1;1.4] | 1.2[1.1;1.2] | 1.0[-1.4;1.4] | -1.5[-2.1;-1.3] | 1.2[-1.0;1.4] |
|  | LPL | lipoprotein lipase | -1.1[-1.3;1.1] | -1.2[-1.3;1.1] | -1.2[-1.3;-1.0] | -1.5[-18.2;-1.0] | -1.8[-10.6;-1.2] | -1.3[-29.0;-1.1] | -1.1[-1.5;1.4] | -1.2[-1.5;1.3] | -1.6[-5.6;1.2] | -1.2[-1.4;1.1] |
|  | PCTP | phosphatidylcholine transfer protein | 1.2[1.1;1.3] | 1.2[-1.1;1.4] | 1.3[1.2;1.4] | 1.4[1.4;1.6] | 1.4[1.3;1.6] | -1.5[-2.1;-1.0] | 1.6[1.3;1.8] | 1.3[1.0;1.8] | -1.3[-2.6;1.3] | 1.3[-1.1;1.7] |
|  | PLTP | phospholipid transfer protein | -1.1[-1.4;-1.1] | -1.1[-1.3;-1.0] | -1.3[-1.4;-1.1] | -1.9[-10.8;-1.3] | -2.0[-9.9;-1.3] | -3.1[-12.1;-2.0] | -1.6[-2.6;-1.8] | -2.0[-3.1;-2.1] | -2.3[-4.6;-2.2] | -1.3[-2.3;-1.1] |
|  | MTTP | microsomal triglyceride transfer protein | -1.0[-1.3;1.3] | -1.1[-1.6;1.2] | 1.1[1.1;1.1] | -1.1[-1.1;1.1] | -1.2[-1.6;-1.1] | -2.0[-2.2;-1.5] | 1.1[-1.0;1.3] | -1.5[-2.1;-1.0] | -2.7[-5.8;-1.6] | 1.1[-1.1;1.2] |
|  | **VLDLR** | **very low density lipoprotein receptor** | **1.1[1.1;1.4]** | **1.2[1.2;1.6]** | **1.3[1.4;1.8]** | **1.7[1.8;2.5]** | **2.3[1.9;4.0]** | **3.4[2.6;5.1]** | **1.3[1.1;1.8]** | **1.9[1.6;4.0]** | **2.7[1.3;4.8]** | **1.5[1.3;2.3]** |
|  | ANGPTL3 | angiopoietin-like 3 | 1.0[-1.4;1.0] | -1.1[-1.9;-1.1] | -1.4[-2.1;-1.4] | -1.2[-2.2;-1.2] | -1.4[-3.8;-1.5] | -2.5[-5.3;-2.0] | 1.2[-1.1;1.3] | -1.2[-2.2;-1.1] | -2.1[-5.8;-2.3] | 1.2[-1.1;1.6] |
| Cholesterol/  Bile tansport and metabolism | **ABCB11** | **ATP-binding cassette, sub-family B (MDR/TAP), member 11** | **-1.2[-1.6;1.2]** | **-1.4[-2.5;1.1]** | **-1.1[1.0;1.1]** | **-1.2[-1.5;1.3]** | **-1.0[-1.6;1.4]** | **-1.1[-1.3;1.5]** | **-1.4[-2.0;-1.0]** | **-2.4[-5.8;1.0]** | **-4.5[-9.9;-1.7]** | **-2.2[-3.3;-1.4]** |
|  | **ABCB4** | **ATP-binding cassette, sub-family B (MDR/TAP), member 4** | **1.5[1.2;2.3]** | **1.6[1.3;2.1]** | **1.8[2.1;2.1]** | **2.1[2.2;3.0]** | **2.2[2.0;3.0]** | **1.2[1.1;2.1]** | **2.4[2.4;3.1]** | **2.6[2.0;3.6]** | **1.8[1.4;2.9]** | **2.0[1.7;3.0]** |
|  | **ABCG5** | **ATP-binding cassette, sub-family G (WHITE), member 5 (sterolin 1)** | **-1.1[-1.5;1.1]** | **-1.2[-1.5;1.0]** | **-1.0[1.0;1.0]** | **-1.1[-1.2;1.1]** | **-1.2[-1.6;-1.1]** | **-2.3[-4.3;-1.4]** | **-1.0[-1.2;1.2]** | **-1.6[-2.6;-1.2]** | **-2.3[-3.5;-1.9]** | **-1.0[-1.4;1.1]** |
|  | **ABCG8** | **ATP-binding cassette, sub-family G (WHITE), member 8 (sterolin 2)** | **1.2[1.1;1.5]** | **1.2[-1.1;1.5]** | **1.2[1.1;1.4]** | **-1.1[-1.3;1.2]** | **-1.3[-1.6;-1.1]** | **-3.2[-5.2;-2.1]** | **-1.1[-1.6;1.6]** | **-1.9[-4.5;-1.2]** | **-3.2[-40.1;-2.1]** | **-1.1[-2.1;1.3]** |
|  | **CYP27A1** | **cytochrome P450, family 27, subfamily A, polypeptide 1** | **-1.1[-1.3;1.1]** | **-1.1[-1.2;1.1]** | **-1.1[-1.1;-1.0]** | **-1.4[-1.4;-1.2]** | **-1.4[-1.8;-1.3]** | **-1.7[-2.0;-1.4]** | **-1.5[-3.0;1.1]** | **-2.4[-4.3;-2.3]** | **-4.0[-8.2;-3.8]** | **-1.6[-3.0;1.0]** |
|  | **CYP7A1** | **cytochrome P450, family 7, subfamily A, polypeptide 1** | **-1.5[-5.5;1.3]** | **-1.6[-8.6;-1.1]** | **-1.8[-1.7;1.2]** | **-1.4[-4.0;1.4]** | **-1.7[-5.2;1.3]** | **-4.8[-13.4;-1.1]** | **-1.4[-3.0;1.2]** | **-3.6[-49.6;1.1]** | **-4.9[-49.0;1.0]** | **-1.3[-3.3;-1.6]** |
|  | **CYP8B1** | **cytochrome P450, family 8, subfamily B, polypeptide 1** | **1.0[-1.1;1.1]** | **-1.1[-1.3;1.0]** | **-1.1[-1.1;1.3]** | **-1.0[1.0;1.3]** | **-1.0[-1.4;1.1]** | **-1.9[-2.1;-1.5]** | **-1.0[-1.1;1.0]** | **-1.8[-5.2;-1.1]** | **-5.6[-100.0;-4.0]** | **1.2[-1.1;1.6]** |
|  | NPC1 | Niemann-Pick disease, type C1 | 1.1[-1.0;1.2] | 1.2[1.0;1.4] | 1.1[1.1;1.2] | 1.2[-1.0;1.5] | 1.3[-1.0;1.6] | 1.9[1.7;2.1] | 1.2[1.0;1.4] | 1.4[1.1;1.8] | 1.4[-1.2;1.9] | 1.3[1.0;1.6] |
|  | RAB9A | RAB9A, member RAS oncogene family | -1.2[-1.2;-1.1] | -1.1[-1.2;-1.0] | -1.2[-1.2;-1.2] | -1.1[-1.1;1.0] | -1.0[-1.0;1.0] | 1.5[1.1;1.9] | -1.0[-1.2;1.1] | -1.1[-1.3;1.2] | 1.1[-1.3;1.6] | 1.0[-1.1;1.2] |
|  | **SLC10A1** | **solute carrier family 10 member 1** | **-1.0[-1.1;1.1]** | **-1.2[-1.3;-1.2]** | **-1.6[-1.8;-1.2]** | **-2.1[-2.7;-1.9]** | **-3.0[-8.2;-2.4]** | **-7.8[-14.5;-6.6]** | **-1.2[-1.4;-1.0]** | **-2.4[-6.9;-1.5]** | **-8.9[-39.1;-9.1]** | **-1.3[-1.7;1.0**] |
|  | SLC10A2 | solute carrier family 10 (sodium/bile acid cotransporter family), member 2 | 1.2[-1.3;1.8] | 1.0[-1.1;1.1] | -1.3[-1.4;1.0] | 5.6[1.0;9.4] | 2.2[-1.6;4.9] | 2.1[-1.2;4.1] | -1.2[-1.3;1.1] | 9.7[1.0;18.2] | 1.4[-1.2;3.1] | -1.1[-1.3;1.3] |
|  | NR1H4 | nuclear receptor subfamily 1, group H, member 4 | 1.2[1.0;1.2] | 1.1[-1.0;1.2] | 1.0[1.1;1.2] | -1.0[-1.3;1.1] | 1.3[-1.5;1.2] | 1.6[-2.0;1.3] | -1.0[1.1;1.8] | -1.4[-1.2;1.4] | -1.0[-1.9;-1.1] | -1.5[-1.1;1.7] |
|  | **CAV1** | **caveolin 1, caveolae protein, 22kDa** | **-1.2[-1.4;1.0]** | **-1.6[-1.6;-1.0]** | **-1.3[-1.8;-1.1]** | **-1.6[-1.6;-1.1]** | **-1.8[-2.0;-1.4]** | **-1.9[-1.5;-1.1]** | **-1.0[-1.6;1.0]** | **-2.4[-1.9;-1.1]** | **-2.8[-5.0;-1.5]** | **-2.6[-1.4;1.0]** |
|  | **ABCC2** | **ATP-binding cassette, sub-family C (CFTR/MRP), member 2** | **1.1[-1.1;1.2]** | **1.2[1.0;1.3]** | **1.4[1.4;1.5]** | **1.6[1.5;1.6]** | **1.8[1.5;2.1]** | **1.7[1.5;2.0]** | **1.1[1.0;1.2]** | **1.5[1.2;1.9]** | **1.7[1.3;2.3]** | **1.3[1.2;1.4]** |
|  | SLCO1B3 | solute carrier organic anion transporter family, member 1B3 | -1.5[-2.6;-1.2] | -1.7[-3.3;-1.5] | -2.0[-2.1;-1.7] | -1.6[-2.1;-1.1] | -1.4[-2.1;-1.2] | -1.9[-3.1;-1.0] | -1.1[-1.4;1.1] | -1.0[-1.4;1.5] | -1.1[-2.5;1.4] | **-1.2[-1.8;-1.0]** |
| Glucose/glycerol tranport and metabolism | **AQP3** | **aquaporin 3 (Gill blood group)** | **1.1[-1.2;2.5]** | **1.3[-1.2;2.4]** | **-1.0[-1.0;1.2]** | **-1.4[-1.8;1.1]** | **-1.4[-2.1;1.5]** | **1.5[1.2;1.8]** | **-1.1[-1.6;1.6]** | **1.1[-1.9;1.9]** | **-1.4[-2.4;1.1]** | **-1.2[-1.9;1.3]** |
|  | AQP7 | aquaporin 7 | 1.1[-1.2;1.5] | 1.2[-1.1;1.5] | 1.5[1.5;1.7] | 1.3[1.2;1.7] | 1.6[1.4;2.1] | 1.5[1.4;2.5] | 1.5[1.3;2.3] | 2.0[1.3;2.9] | 1.8[1.5;2.9] | 1.5[1.3;2.3] |
|  | AQP9 | aquaporin 9 | -1.1[-1.4;1.1] | -1.2[-1.6;1.1] | 1.1[-1.0;1.1] | -1.1[-1.7;1.0] | -1.1[-1.5;1.1] | 1.1[-1.5;1.4] | -1.2[-1.6;-1.0] | -1.4[-1.9;1.0] | -1.3[-2.9;1.3] | -1.4[-1.7;-1.1] |
|  | **FBP2** | **fructose-1,6-bisphosphatase 2** | **1.0[-1.1;1.2]** | **1.5[-1.1;3.3]** | **1.0[1.0;1.0]** | **1.0[1.0;1.0]** | **8.3[1.0;12.7]** | **1.8[1.2;2.5]** | **4.3[-1.0;7.4]** | **1.0[-1.1;1.0]** | **1.0[-1.0;1.1]** | **15.4[1.0;26.6]** |
|  | **G6PC** | **glucose-6-phosphatase, catalytic subunit** | **-1.4[-1.8;1.0]** | **-1.1[-1.9;1.5]** | **-1.1[-1.1;1.4]** | **-1.2[-1.6;1.5]** | **-1.2[-2.5;1.2]** | **-1.6[-1.6;1.3]** | **1.3[1.2;1.8]** | **1.7[1.2;4.1]** | **3.1[1.4;63.3]** | **1.4[1.4;1.9]** |
|  | **GK** | **glycerol kinase** | **1.1[1.0;1.2]** | **1.1[-1.2;1.3]** | **1.3[1.3;1.3]** | **1.3[1.2;1.4]** | **1.3[1.1;1.4]** | **1.6[1.2;2.2]** | **1.9[1.8;2.2]** | **2.3[2.1;2.8]** | **3.7[2.7;5.0]** | **1.9[1.7;2.0]** |
|  | GPD1 | glycerol-3-phosphate dehydrogenase 1 (soluble) | 1.1[-1.1;1.3] | 1.2[-1.1;1.6] | 1.1[1.1;1.2] | -1.0[-1.1;1.3] | -1.0[-1.5;1.3] | -1.7[-1.9;-1.4] | -1.1[-1.5;1.5] | -1.5[-2.0;-1.4] | -3.2[-6.2;-2.8] | 1.0[-1.4;1.4] |
|  | GPD2 | glycerol-3-phosphate dehydrogenase 2 (mitochondrial) | 1.1[-1.2;1.3] | -1.1[-1.2;1.0] | 1.0[-1.2;1.2] | 1.0[-1.3;1.3] | -1.2[-1.6;1.0] | -1.0[-1.5;1.1] | 1.2[-1.2;1.7] | 1.2[-1.2;1.5] | 1.3[1.1;1.4] | 1.1[1.0;1.2] |
|  | **GYS1** | **glycogen synthase 1 (muscle)** | **1.6[-1.2;3.1]** | **1.5[-1.1;3.6]** | **1.0[-1.3;1.4]** | **-1.2[-1.9;1.1]** | **-1.2[-1.6;1.1]** | **-1.9[-2.8;-1.5]** | **-1.0[-1.2;1.3]** | **1.0[-1.3;1.5]** | **-1.3[-1.8;-1.1]** | **-1.1[-1.3;1.1]** |
|  | **GYS2** | **glycogen synthase 2 (liver)** | **-1.7[-2.9;-1.1]** | **-1.9[-3.7;-1.4]** | **-1.9[-1.8;-1.7]** | **-1.6[-1.9;-1.3]** | **-1.7[-2.5;-1.4]** | **-3.4[-5.5;-1.8]** | **-1.2[-1.3;1.2]** | **-2.0[-5.8;-1.3]** | **-3.2[-8.7;-1.8]** | **-1.3[-1.8;-1.3]** |
|  | LDHA | lactate dehydrogenase A | -1.1[-1.0;1.2] | -1.1[-1.0;1.2] | -1.1[-1.0;1.1] | -1.0[-1.1;1.5] | -1.2[-1.4;1.4] | -1.2[-1.6;-1.0] | -1.0[1.0;1.2] | -1.1[-1.2;1.2] | -1.5[-2.3;1.2] | 1.0[-1.1;1.3] |
|  | PCK1 | phosphoenolpyruvate carboxykinase 1 (soluble) | -1.1[-1.9;1.3] | -1.0[-1.8;1.3] | 1.2[1.1;1.6] | 1.0[1.0;1.8] | 1.0[-1.1;1.8] | 2.2[1.5;9.9] | 1.2[-1.0;4.0] | 1.1[1.2;1.9] | 1.2[-1.7;4.9] | 1.2[-1.1;2.5] |
|  | **PDK4** | **pyruvate dehydrogenase kinase, isozyme 4** | **-1.1[-2.3;2.1]** | **1.1[-2.3;3.2]** | **2.1[1.8;3.6]** | **2.3[2.7;4.3]** | **3.4[2.5;9.5]** | **8.9[5.7;33.8]** | **6.4[5.8;11.8]** | **10.8[6.0;25.5]** | **20.0[5.4;72.4]** | **6.5[5.5;11.1]** |
| Biotransformation | **AKR1B10** | **aldo-keto reductase family 1, member B10 (aldose reductase)** | **1.3[1.2;2.3]** | **1.6[1.4;3.3]** | **1.2[1.7;1.9]** | **1.9[1.8;34.3]** | **2.6[1.5;39.3]** | **1.3[-1.8;21.3]** | **1.0[1.1;1.3]** | **1.5[1.1;3.9]** | **1.1[-5.1;4.6]** | **1.3[1.2;2.5]** |
|  | AKR1C3 | aldo-keto reductase family 1, member C3 | 1.1[-1.0;1.6] | 1.2[-1.0;1.6] | 1.1[1.1;1.3] | 1.4[1.2;2.4] | 1.5[1.2;2.5] | 1.3[-1.0;2.2] | 1.2[1.0;1.6] | 1.4[1.2;1.9] | 1.3[1.1;2.0] | 1.3[1.2;1.5] |
|  | **CYP1A2** | **cytochrome P450, family 1, subfamily A, polypeptide 2** | **-1.1[-1.9;1.4]** | **1.1[-1.1;1.5]** | **1.4[1.5;1.6]** | **1.6[2.0;5.2]** | **2.4[2.4;11.7]** | **1.2[-1.0;5.0]** | **-1.3[-2.5;-1.3]** | **-2.0[-4.6;-2.1]** | **-4.0[-11.9;-2.0]** | **-1.5[-2.8;-1.4]** |
|  | **CYP2B6** | **cytochrome P450, family 2, subfamily B, polypeptide 6** | **1.5[1.2;2.1]** | **1.9[1.4;4.2]** | **2.0[1.5;2.6]** | **2.5[1.8;4.1]** | **2.4[1.5;4.6]** | **1.4[1.3;2.7]** | **-1.1[-1.3;-1.0]** | **-1.1[-1.4;-1.1]** | **-1.5[-1.9;-1.5]** | **1.4[1.1;1.5]** |
|  | **CYP2C8** | **cytochrome P450, family 2, subfamily C, polypeptide 8** | **1.4[1.3;4.1]** | **1.5[-1.0;4.2]** | **2.7[3.1;4.4]** | **1.6[2.0;4.7]** | **1.5[1.8;2.2]** | **-1.9[-3.5;1.1]** | **1.2[1.1;2.0]** | **-1.5[-5.8;-1.2]** | **-3.0[-48.4;-6.4]** | **1.3[1.0;2.5]** |
|  | CYP2C9 | cytochrome P450, family 2, subfamily C, polypeptide 9 | 1.3[-1.0;1.9] | 1.3[-1.1;1.9] | 1.8[1.8;1.9] | 1.2[1.1;1.5] | 1.1[-1.1;1.2] | -1.8[-2.5;-1.2] | -1.1[-1.5;-1.2] | -1.4[-2.1;-1.4] | -1.8[-3.6;-2.1] | 1.0[-1.4;1.2] |
|  | **CYP2J2** | **cytochrome P450, family 2, subfamily J, polypeptide 2** | **1.1[-1.0;1.2]** | **1.0[-1.0;1.1]** | **1.1[1.1;1.1]** | **1.1[-1.0;1.5]** | **1.1[-1.1;1.4]** | **-1.3[-1.4;-1.0]** | **1.5[1.1;2.1]** | **1.4[1.2;1.7]** | **-1.1[-1.5;1.4]** | **1.4[1.1;1.8]** |
|  | CYP3A5 | cytochrome P450, family 3, subfamily A, polypeptide 5 | 1.0[-1.2;1.3] | 1.1[-1.2;1.4] | 1.6[1.5;1.7] | 1.4[1.2;1.7] | 1.6[1.1;1.8] | 2.6[1.9;2.5] | 1.1[-1.1;1.1] | 1.1[-1.1;1.2] | 1.0[-1.2;1.1] | 1.1[-1.3;1.6] |
|  | **CYP3A7** | **cytochrome P450, family 3, subfamily A, polypeptide 4** | **1.5[-1.2;3.1]** | **1.6[-1.2;3.4]** | **2.7[2.1;3.3]** | **1.8[1.1;3.0]** | **1.8[1.0;2.5]** | **-1.1[-1.2;1.7]** | **1.2[-1.1;1.5]** | **1.0[-1.3;1.1]** | **-1.3[-2.0;-1.2]** | **1.2[-1.4;1.9]** |
|  | **CYP3A4** | **cytochrome P450, family 3, subfamily A, polypeptide 7** | **1.5[-1.1;4.8]** | **1.7[-1.2;5.5]** | **3.4[4.0;5.4]** | **1.6[1.1;4.7]** | **1.6[1.1;2.4]** | **-1.3[-1.7;1.7]** | **-1.0[-1.5;1.1]** | **-1.2[-1.7;-1.1]** | **-1.5[-4.1;-1.4]** | **1.1[-1.4;1.6]** |
|  | **EPHX2** | **epoxide hydrolase 2, cytoplasmic** | **-1.0[-1.3;1.1]** | **-1.1[-1.5;1.0]** | **-1.1[-1.1;1.2]** | **-1.4[-1.6;-1.1]** | **-1.5[-2.4;-1.4]** | **-2.5[-4.2;-1.6]** | **-1.2[-1.4;-1.2]** | **-1.9[-5.0;-1.6]** | **-3.3[-9.3;-3.9]** | **-1.3[-1.8;-1.3]** |
|  | **GSTA3** | **glutathione S-transferase A3** | **-1.1[-1.3;1.0]** | **-1.0[-1.4;1.3]** | **-1.1[-1.4;1.2]** | **1.2[-1.3;1.5]** | **1.4[-1.3;1.8]** | **1.3[1.3;2.1]** | **-1.3[-2.1;1.1]** | **-2.0[-3.2;-1.7]** | **-5.4[-8.2;-3.8]** | **-1.1[-2.2;1.5]** |
|  | **MGST3** | **microsomal glutathione S-transferase 3** | **1.2[1.1;1.4]** | **1.2[1.1;1.7]** | **1.2[1.2;1.4]** | **1.2[1.2;1.7]** | **1.3[1.3;1.6]** | **1.2[1.1;1.7]** | **1.4[1.4;1.7]** | **1.5[1.4;1.8]** | **1.1[-1.3;1.7]** | 1.5[1.4;1.9] |
|  | CYP2E1 | cytochrome P450, family 2, subfamily E, polypeptide 1 | -1.0[-1.9;1.1] | -1.1[-2.9;1.2] | 1.5[1.2;1.5] | -1.1[-2.7;-1.0] | -1.2[-3.3;1.2] | -1.5[-2.5;1.5] | 1.0[-1.7;-1.0] | -1.3[-2.8;1.1] | -1.5[-8.0;1.3] | -1.1[-2.3;-1.1] |
|  | **CYP1A1** | **cytochrome P450, family 1, subfamily A, polypeptide 1** | **-1.0[-1.1;1.4]** | **1.0[-1.2;2.1]** | **1.1[1.3;1.4]** | **3.8[3.3;7.6]** | **5.9[3.3;45.0]** | **5.8[4.8;34.4]** | **-1.4[-1.7;1.2]** | **-1.9[-1.2;-2.2]** | **-2.0[-1.4;-4.0]** | **-1.5[-1.2;-2.2]** |
| Amino acid metabolism | **ABAT** | **4-aminobutyrate aminotransferase** | **-1.1[-1.4;-1.1]** | **-1.2[-1.5;-1.2]** | **-1.3[-1.4;-1.2]** | **-1.6[-2.7;-1.5]** | **-1.8[-3.6;-1.7]** | **-2.2[-3.6;-2.0]** | **-1.6[-2.1;-1.8]** | **-2.5[-5.4;-2.7]** | **-4.4[-7.7;-4.7]** | **-1.7[-2.5;-1.8]** |
|  | **ACMSD** | **aminocarboxymuconate semialdehyde decarboxylase** | **-1.1[-1.3;-1.0]** | **-1.3[-1.9;-1.1]** | **-1.3[-1.3;-1.0]** | **-1.3[-1.3;-1.1]** | **-1.4[-2.4;-1.2]** | **-2.9[-4.5;-2.0]** | **-1.1[-1.1;1.0]** | **-1.3[-2.0;-1.2]** | **-1.8[-2.9;-1.4]** | **-1.1[-1.3;1.1]** |
|  | **AGXT2** | **alanine-glyoxylate aminotransferase 2** | **-1.2[-1.6;-1.0]** | **-1.3[-2.1;-1.0]** | **-1.5[-1.6;-1.0]** | **-1.5[-1.6;-1.2]** | **-1.8[-4.4;-1.6]** | **-3.2[-4.5;-2.6]** | **-1.3[-1.5;-1.2]** | **-2.5[-6.5;-2.0]** | **-4.3[-9.9;-5.0]** | **-1.4[-1.7;-1.3]** |
|  | **ARG1** | **arginase, liver** | **-1.1[-1.5;1.1]** | **-1.1[-2.0;1.2]** | **-1.1[1.1;1.1]** | **-1.3[-1.2;1.0]** | **-1.4[-3.7;-1.2]** | **-2.4[-4.4;-2.6]** | **-1.3[-1.7;-1.4]** | **-2.3[-10.9;-2.0]** | **-4.9[-14.2;-9.3]** | **-1.3[-2.0;-1.5]** |
|  | ASL | argininosuccinate lyase | 1.0[-1.0;1.2] | 1.0[-1.1;1.2] | 1.0[-1.0;1.1] | -1.2[-1.6;1.0] | -1.2[-1.5;1.0] | -1.3[-1.8;-1.1] | -1.2[-1.2;-1.1] | -1.3[-1.6;-1.1] | -1.6[-2.3;-1.4] | -1.3[-1.4;-1.2] |
|  | ASS1 | argininosuccinate synthetase 1 | 1.2[-1.3;1.6] | 1.3[-1.2;1.7] | 1.3[1.1;1.3] | -1.2[-1.7;1.2] | -1.4[-2.5;1.1] | -1.2[-1.6;-1.1] | -1.4[-2.5;1.2] | -1.3[-1.7;-1.1] | -2.2[-4.3;-1.6] | -1.3[-3.4;1.1] |
|  | CBS | cystathionine-beta-synthase | 1.0[-1.1;1.1] | -1.0[-1.1;1.1] | -1.0[-1.2;1.0] | -1.3[-1.5;-1.2] | -1.3[-1.5;-1.3] | -1.7[-2.2;-1.5] | -1.6[-2.1;-1.5] | -1.7[-2.2;-1.4] | -2.1[-2.6;-2.2] | -1.7[-2.1;-1.9] |
|  | CTH | cystathionase (cystathionine gamma-lyase) | -1.4[-1.8;-1.0] | -1.4[-2.1;-1.1] | -1.2[-1.1;-1.1] | -1.5[-2.5;-1.2] | -1.2[-1.8;1.3] | 1.9[1.8;2.5] | -1.4[-2.0;1.1] | 1.3[-2.1;2.8] | 2.7[-1.1;7.4] | -1.9[-3.0;-1.4] |
|  | GLS | glutaminase | 1.2[-1.2;1.2] | 1.1[-1.1;1.2] | 1.2[1.1;1.2] | 1.2[-1.2;1.2] | 1.3[-1.1;1.2] | -1.1[-1.4;1.2] | 1.2[-1.4;1.3] | 1.6[1.0;1.8] | 2.4[1.8;3.0] | 1.2[-1.2;1.4] |
|  | **GLS2** | **glutaminase 2 (liver, mitochondrial)** | **-1.1[-1.4;1.0]** | **-1.2[-1.8;1.0]** | **1.0[-1.1;1.1]** | **-1.6[-5.4;-1.3]** | **-1.7[-6.2;-1.3]** | **-2.1[-7.2;-1.2]** | **-1.4[-2.3;-1.5]** | **-1.9[-3.5;-1.9]** | **-3.2[-10.9;-2.3]** | **-1.7[-2.9;-1.6]** |
|  | **GOT2** | **glutamic-oxaloacetic transaminase 2, mitochondrial** | **1.3[1.0;1.6]** | **1.3[1.1;1.7]** | **1.3[1.3;1.6]** | **-1.0[-1.1;1.3]** | **-1.0[-1.1;1.1]** | **-1.9[-2.3;-1.3]** | **1.1[-1.1;1.4]** | **-1.1[-1.2;1.3]** | **-1.7[-2.7;-1.1]** | **1.2[-1.1;1.6]** |
|  | GPT | glutamic-pyruvate transaminase (alanine aminotransferase) | 1.4[-1.5;4.5] | 1.5[-1.6;4.1] | 1.0[-1.3;1.6] | 1.0[-1.3;1.4] | 1.0[-1.0;1.2] | 1.0[-1.4;1.0] | 1.0[-1.6;1.6] | 1.1[-1.4;1.7] | -1.0[-1.4;1.2] | 1.1[-1.4;1.6] |
|  | HAL | histidine ammonia-lyase | -1.3[-1.4;1.1] | -1.5[-1.5;-1.3] | -1.4[-1.4;-1.3] | -1.4[-2.4;1.0] | -2.0[-3.2;-1.1] | -3.6[-6.8;-3.3] | -1.1[-1.2;1.1] | -1.3[-1.4;1.4] | -2.1[-4.1;-1.1] | -1.1[-1.4;1.4] |
|  | HPD | 4-hydroxyphenylpyruvate dioxygenase | -1.0[-1.2;1.1] | -1.1[-1.4;1.0] | -1.1[-1.3;-1.1] | -1.5[-1.7;-1.3] | -1.6[-2.1;-1.4] | -2.2[-2.4;-2.1] | -1.4[-1.9;-1.1] | -1.8[-2.5;-1.6] | -2.9[-4.7;-3.1] | -1.3[-2.3;-1.2] |
|  | OAT | ornithine aminotransferase (gyrate atrophy) | -1.3[-2.2;1.1] | -1.4[-2.1;1.1] | -1.1[-1.2;-1.1] | -1.0[-1.5;1.1] | -1.2[-1.8;1.0] | 1.0[-1.0;1.2] | 1.0[-1.7;1.5] | 1.2[-1.8;1.7] | 2.3[1.2;5.4] | -1.1[-2.0;1.4] |
|  | ODC1 | ornithine decarboxylase 1 | 1.1[-1.0;1.5] | 1.1[-1.1;1.3] | 1.1[-1.1;1.1] | 1.0[-1.1;1.0] | 1.0[-1.1;1.2] | 1.2[1.0;1.3] | -1.1[-1.3;1.1] | 1.2[1.0;1.5] | 1.6[1.2;2.7] | -1.0[-1.0;1.0] |
|  | **OTC** | **ornithine carbamoyltransferase** | **-1.2[-1.4;-1.2]** | **-1.3[-1.8;-1.3]** | **-1.7[-2.0;-1.2]** | **-1.7[-3.6;-1.6]** | **-2.0[-7.8;-1.8]** | **-5.4[-13.1;-3.8]** | **-1.5[-2.7;-1.8]** | **-2.4[-12.1;-2.4]** | **-5.2[-23.2;-4.2]** | **-1.4[-2.5;-1.7]** |
|  | **PAH** | **phenylalanine hydroxylase** | **-1.0[-1.2;1.3]** | **-1.1[-1.8;1.2]** | **-1.0[1.2;1.3]** | **-1.2[-1.3;1.2]** | **-1.2[-1.7;1.1]** | **-2.0[-2.5;-1.1]** | **-1.1[-1.4;1.3]** | **-1.3[-2.3;-1.2]** | **-1.6[-2.5;-1.5]** | **-1.2[-1.7;1.1]** |
|  | **PSAT1** | **phosphoserine aminotransferase 1** | **-1.1[-1.3;1.0]** | **-1.1[-1.3;-1.0]** | **-1.2[-1.2;-1.0]** | **-1.0[-1.1;1.1]** | **1.2[-1.0;1.4]** | **1.8[1.6;3.5]** | **1.1[-1.1;1.3]** | **2.1[1.5;3.7]** | **3.2[2.7;4.1]** | **1.0[-1.2;1.2]** |
|  | **TAT** | **tyrosine aminotransferase** | **-1.3[-2.0;-1.2]** | **-1.5[-3.1;-1.4]** | **-1.6[-1.9;-1.4]** | **-1.8[-4.5;-1.8]** | **-2.3[-8.2;-2.0]** | **-2.5[-5.9;-2.8]** | **-1.4[-2.1;-1.6]** | **-1.6[-5.7;-1.8]** | **-2.4[-10.1;-2.6]** | **-1.8[-3.0;-2.4]** |
| Inflammation | APCS | amyloid P component, serum | -1.3[-1.7;-1.1] | -1.2[-1.7;1.1] | -1.0[-1.1;-1.0] | -1.1[-1.2;1.0] | -1.4[-1.8;-1.3] | -1.8[-2.4;-1.5] | -1.3[-1.9;1.1] | -2.3[-3.4;-2.2] | -7.4[-15.2;-5.0] | -1.2[-1.5;-1.1] |
|  | **BIRC3** | **baculoviral IAP repeat-containing 3** | **-1.1[-1.3;1.1]** | **-1.2[-1.4;1.0]** | **-1.1[-1.4;-1.1]** | **-1.1[-2.0;-1.1]** | **-1.4[-1.8;-1.3]** | **-1.3[-2.9;-1.4]** | **-1.1[-1.7;1.0]** | **-1.2[-1.4;1.0]** | **-1.3[-3.3;1.0]** | **-1.2[-1.4;1.0**] |
|  | **CD68** | **CD68 molecule** | **1.4[-1.3;2.3]** | **1.5[-1.1;2.6]** | **1.3[1.3;1.6]** | **-1.2[-1.3;1.2]** | **-1.0[-1.4;1.3]** | **-1.1[-1.2;1.1]** | **-1.4[-1.9;-1.1]** | **-1.4[-1.6;-1.2]** | **-1.8[-2.1;-1.4]** | **-1.5[-2.4;1.0]** |
|  | CEBPB | CCAAT/enhancer binding protein (C/EBP), beta | -1.1[-1.2;-1.1] | -1.1[-1.3;-1.0] | -1.1[-1.2;-1.1] | -1.3[-1.3;-1.0] | -1.3[-1.5;-1.1] | 1.3[-1.0;1.5] | -1.5[-1.7;-1.2] | -1.6[-1.9;-1.1] | -1.3[-2.1;1.6] | -1.5[-1.8;-1.3] |
|  | CRP | C-reactive protein, pentraxin-related | -1.1[-2.0;1.2] | -1.1[-1.8;1.2] | 1.3[1.2;1.4] | 1.1[-1.0;1.5] | 1.2[1.1;1.9] | 2.6[2.1;8.5] | 1.0[-1.5;2.6] | -1.0[-1.6;4.0] | 1.0[-1.5;3.9] | -1.1[-1.8;1.6] |
|  | CXCL10 | chemokine (C-X-C motif) ligand 10 | -1.2[-1.4;-1.0] | -1.3[-1.6;-1.3] | -1.4[-2.0;-1.4] | -1.9[-16.9;-1.3] | -4.6[-42.9;-1.7] | -6.3[-74.6;-7.0] | -1.5[-1.9;-1.3] | -4.1[-7.3;-4.6] | -20.3[-100.0;-8.7] | -1.3[-2.1;1.6] |
|  | EMR1 | egf-like module containing, mucin-like, hormone receptor-like 1 | -1.1[-5.2;1.3] | -1.2[-2.1;1.1] | 1.1[-1.0;1.5] | -1.8[-7.0;1.1] | -1.4[-5.3;1.8] | -1.9[-4.3;-1.3] | -2.4[-9.2;-1.4] | -3.8[-5.6;-2.3] | -2.5[-7.7;-1.7] | -2.1[-4.1;-1.7] |
|  | FGB | fibrinogen beta chain | -1.0[-1.3;1.1] | -1.1[-1.7;1.1] | 1.1[-1.0;1.2] | -1.1[-1.5;1.0] | -1.5[-2.8;-1.2] | -1.6[-2.1;-1.3] | 1.1[-1.2;1.4] | -1.3[-1.8;-1.1] | -1.6[-2.4;-1.5] | -1.0[-1.7;1.4] |
|  | **ICAM1** | **intercellular adhesion molecule 1 (CD54),** | **-1.0[-1.1;1.1]** | **-1.1[-1.0;1.0]** | **-1.0[-1.2;-1.0]** | **-1.1[-1.9;-1.1]** | **-1.2[-1.4;1.0]** | **1.5[-1.4;2.2]** | **-1.3[-1.5;-1.3]** | **-1.7[-2.4;-1.6]** | **-2.0[-4.6;-1.5]** | **-1.3[-1.6;-1.2]** |
|  | NFKB1 | nuclear factor of kappa light polypeptide gene enhancer in B-cells 1 | 1.0[-1.1;1.1] | 1.0[-1.0;1.2] | 1.0[-1.1;1.1] | -1.1[-1.4;-1.1] | -1.2[-1.4;-1.2] | -1.4[-1.9;-1.4] | -1.2[-1.2;-1.1] | -1.4[-1.6;-1.3] | -1.6[-2.3;-1.4] | -1.1[-1.2;-1.0] |
|  | NFKB2 | nuclear factor of kappa light polypeptide gene enhancer in B-cells 2 ) | 1.0[-1.2;1.3] | 1.1[-1.2;1.5] | -1.2[-1.4;-1.3] | -1.3[-1.7;-1.1] | -1.4[-2.0;1.0] | 1.0[-1.4;1.1] | -1.6[-2.4;-1.2] | -1.8[-2.7;-1.4] | -1.4[-2.3;1.1] | -1.3[-2.3;1.2] |
|  | IL1B | interleukin 1, beta | 1.0[-1.2;1.3] | -1.0[-1.5;1.1] | 1.3[1.2;1.3] | 1.5[-1.4;1.9] | 1.3[-1.5;3.1] | 2.0[-1.2;3.0] | 1.3[-1.1;1.6] | 1.4[-1.3;2.8] | 1.4[-1.5;3.2] | 1.1[-1.2;2.0] |
|  | IL1R1 | interleukin 1 receptor, type I | -1.1[-1.5;-1.0] | -1.2[-1.8;-1.0] | -1.1[-1.4;1.0] | -1.0[-1.2;1.1] | -1.0[-1.3;1.0] | 1.4[-1.1;2.5] | 1.0[-1.2;1.1] | -1.1[-1.4;1.0] | -1.1[-1.5;1.2] | -1.1[-1.2;1.0] |
|  | IL1RAP | interleukin 1 receptor accessory protein | -1.1[-1.3;1.1] | -1.2[-1.5;1.0] | -1.1[-1.1;-1.0] | -1.1[-1.2;1.2] | -1.5[-1.7;-1.3] | -2.4[-1.8;-1.7] | -1.1[-1.2;1.1] | -1.7[-2.4;-1.2] | -2.4[-2.6;-2.0] | -1.1[-1.3;1.2] |
|  | IL1RN | interleukin 1 receptor antagonist | -1.2[-1.3;-1.1] | -1.3[-1.6;1.2] | -1.1[-1.1;1.1] | -1.1[-1.1;1.1] | -1.1[-1.2;1.1] | 1.1[-1.0;2.4] | -1.1[-1.1;1.2] | -1.2[-1.2;-1.0] | -1.1[-1.4;1.3] | -1.1[-1.4;1.4] |
|  | IL6 | interleukin 6 (interferon, beta 2) | 1.0[-3.8;1.2] | -1.0[-1.8;1.3] | -1.0[-1.5;-1.2] | 1.3[1.0;1.2] | 1.1[-1.1;1.1] | 1.2[-1.1;1.7] | 1.2[-1.3;1.3] | -1.0[-2.1;1.4] | 2.0[1.0;4.1] | 1.1[-1.2;1.4] |
|  | IL6R | interleukin 6 receptor | -1.1[-1.1;1.0] | -1.1[-1.4;-1.1] | -1.2[-1.1;-1.0] | -1.2[-1.3;1.2] | -1.0[-1.3;1.2] | 1.3[1.1;1.9] | -1.2[-1.4;1.0] | -1.2[-1.5;1.3] | 1.2[-1.4;2.3] | -1.6[-2.1;-1.2] |
|  | IL8 | interleukin 8 | 1.1[-1.6;1.4] | 1.2[-1.6;1.6] | 1.0[-1.7;1.4] | 1.2[-2.7;1.3] | 1.0[-3.1;-1.2] | 1.4[-2.5;3.2] | 1.4[-2.3;1.7] | 1.0[-1.9;1.1] | 1.3[-4.4;3.8] | 1.1[-3.0;2.2] |
|  | **CCL2** | **chemokine (C-C motif) ligand 2** | **-1.0[-1.3;1.2]** | **-1.0[-1.3;1.2]** | **-1.0[-1.4;-1.0]** | **1.1[-7.3;-1.0]** | **-1.1[-10.0;-1.0]** | **1.1[-18.6;1.1]** | **-1.2[-3.2;-1.0]** | **-1.6[-7.1;-1.6]** | **-3.9[-33.7;-13.7]** | **-1.1[-2.7;1.1]** |
|  | **CCL3** | **chemokine (C-C motif) ligand 3** | **-1.4[-1.5;1.0]** | **-1.7[-1.8;-1.4]** | **-2.0[-1.9;-1.6]** | **-2.1[-24.1;-1.4]** | **-2.5[-22.4;-1.2]** | **-2.1[-50.6;-1.3]** | **-2.1[-2.7;-1.7]** | **-3.2[-5.8;-2.6]** | **-3.0[-12.4;-2.2]** | **-2.0[-3.2;-1.3]** |
|  | LCN2 | lipocalin 2 | 1.1[1.1;1.3] | 1.1[1.0;1.3] | 1.1[1.0;1.3] | -1.1[-2.9;1.1] | -1.1[-2.4;1.1] | -1.1[-3.3;1.1] | 1.0[-1.0;1.2] | -1.1[-1.3;1.0] | -1.1[-1.5;1.1] | -1.1[-1.4;1.1] |
|  | LIFR | leukemia inhibitory factor receptor alpha | -1.0[-1.4;1.1] | 1.1[-1.3;1.7] | -1.1[-1.6;1.1] | 1.3[-1.3;1.6] | 1.3[-1.4;2.3] | 1.9[-1.1;2.6] | 1.3[1.0;1.6] | 1.6[-1.1;2.1] | 2.6[1.5;4.8] | 1.0[-1.2;1.4] |
|  | MT1A | metallothionein 1A | 1.1[-1.1;1.1] | 1.1[-1.1;1.1] | 1.2[-1.0;1.1] | 1.2[-1.0;1.1] | 1.3[1.0;1.5] | 2.1[1.6;2.9] | 1.1[-1.1;1.2] | 1.4[1.1;1.9] | 2.0[1.4;3.4] | -1.1[-1.5;-1.0] |
|  | ORM2 | orosomucoid 2 | -1.1[-1.1;-1.0] | -1.1[-1.2;-1.0] | -1.0[-1.1;1.1] | -1.1[-1.2;1.0] | -1.1[-1.2;-1.0] | -1.1[-1.4;1.0] | -1.0[-1.2;1.1] | -1.1[-1.3;1.0] | -1.5[-1.7;-1.4] | -1.0[-1.1;1.0] |
|  | PLA1A | phospholipase A1 member A | 1.1[-1.2;1.2] | 1.2[-1.0;1.3] | 1.2[1.1;1.4] | 1.5[1.2;3.4] | 2.1[1.6;4.7] | 1.5[1.2;3.0] | 1.6[1.4;3.5] | 1.8[1.5;3.4] | 2.1[1.4;6.5] | 2.0[1.3;3.9] |
|  | SAA2 | serum amyloid A2 | 1.2[-1.1;1.9] | 1.2[-1.1;1.7] | -1.2[-1.2;-1.1] | -1.3[-4.2;1.2] | -1.2[-2.2;1.4] | -1.1[-3.0;1.1] | -1.1[-1.4;1.5] | -1.5[-1.6;-1.1] | -2.0[-2.4;-1.5] | -1.4[-1.6;-1.1] |
|  | **SAA4** | **serum amyloid A4, constitutive** | **-1.1[-1.4;1.3]** | **-1.2[-1.6;1.1]** | **-1.8[-1.9;-1.4]** | **-1.6[-1.7;-1.3]** | **-1.9[-3.8;-1.5]** | **-2.9[-4.6;-1.9]** | **-1.2[-1.4;1.2]** | **-1.9[-5.2;-1.1]** | **-4.8[-28.1;-3.0]** | **-1.2[-1.3;-1.0]** |
|  | STAT2 | signal transducer and activator of transcription 2, 113kDa | 1.0[-1.0;1.2] | -1.0[-1.3;1.2] | -1.2[-1.3;1.0] | -1.3[-1.6;-1.0] | -1.2[-1.8;1.1] | -1.1[-1.7;1.1] | -1.2[-1.4;-1.0] | -1.4[-1.7;-1.3] | -1.3[-1.8;1.1] | -1.2[-1.5;1.0] |
|  | STAT3 | signal transducer and activator of transcription 3 | -1.1[-1.0;1.6] | -1.1[-1.1;1.5] | 1.0[1.1;1.1] | -1.2[-1.4;1.0] | -1.4[-1.5;-1.2] | -1.4[-1.5;-1.2] | -1.1[-1.6;-1.0] | -1.3[-1.8;-1.3] | -1.8[-1.9;-1.6] | -1.0[-1.9;1.0] |
|  | TRAF1 | TNF receptor-associated factor 1 | -1.1[-1.3;1.2] | 1.1[-1.4;1.2] | -1.1[-1.7;-1.2] | 1.1[-1.6;1.0] | -1.1[-1.6;1.1] | 1.2[-1.3;1.4] | -1.4[-2.5;-1.5] | -1.8[-3.1;-2.0] | -1.2[-3.0;1.2] | -1.2[-1.5;-1.1] |
|  | **VCAM1** | **vascular cell adhesion molecule 1** | **-1.0[-1.6;1.2]** | **-1.3[-2.6;-1.1]** | **-1.2[-1.5;-1.3]** | **-1.7[-20.9;-1.1]** | **-2.6[-71.3;-1.5]** | **-2.0[-25.5;-1.2]** | **-1.2[-1.8;-1.1]** | **-3.0[-12.5;-2.8]** | **-6.2[-31.3;-2.8]** | **-1.1[-2.3;1.5]** |
|  | **VNN1** | **vanin 1** | **2.1[1.2;1.5]** | **1.9[1.2;1.5]** | **1.6[1.4;1.4]** | **1.4[1.2;1.8]** | **-1.9[1.1;1.6]** | **-1.3[1.1;1.6]** | **1.4[1.7;2.9]** | **1.5[1.4;2.6]** | **-3.9[1.1;3.7]** | **-6.7[1.4;2.7]** |
|  | **PTGS2** | **prostaglandin-endoperoxide synthase 2** | **1.1[-1.1;1.4]** | **-1.0[-1.2;1.2]** | **1.1[-1.0;1.2]** | **-1.0[-1.4;1.2]** | **1.0[-1.2;1.3]** | **1.2[-1.2;1.6]** | **1.0[-1.1;1.3]** | **1.1[-1.3;1.8]** | **3.0[-1.2;6.2]** | **-1.0[-1.3;1.1]** |
| Oxidative stress/Immune response | **HMOX1** | **heme oxygenase (decycling) 1** | **2.5[1.2;7.6]** | **2.4[1.3;9.7]** | **1.5[-1.0;3.6]** | **1.6[1.3;3.1]** | **2.1[2.1;3.6]** | **1.1[-1.8;1.6]** | **1.7[1.2;3.4]** | **1.8[1.3;3.8]** | **1.5[-1.1;4.3]** | **1.3[-1.2;5.4]** |
|  | **POR** | **P450 (cytochrome) oxidoreductase** | **1.7[1.0;2.2]** | **1.9[1.2;2.3]** | **2.1[1.5;2.5]** | **2.0[1.7;2.5]** | **2.4[1.7;3.1]** | **3.0[2.5;3.6]** | **1.5[-1.2;2.0]** | **2.0[1.6;2.8]** | **2.3[1.8;2.5]** | **1.4[-1.0;1.9]** |
|  | **OASL** | **2'-5'-oligoadenylate synthetase-like** | **1.5[1.2;1.9]** | **1.7[1.5;2.6]** | **1.4[1.6;1.6]** | **1.8[1.5;4.2]** | **2.3[1.7;4.9]** | **3.8[2.7;5.8]** | **2.2[1.7;3.7]** | **3.7[3.6;4.9]** | **8.6[8.8;11.0]** | **2.6[2.0;4.3]** |
|  | **MBL2** | **mannose-binding lectin (protein C) 2, soluble (opsonic defect)** | **1.6[1.6;2.5]** | **1.7[1.6;3.1]** | **1.4[2.1;2.3]** | **1.9[3.4;6.9]** | **1.8[1.4;5.7]** | **-1.9[-5.6;1.3]** | **2.1[2.9;3.9]** | **1.2[1.0;4.0]** | **-2.1[-32.4;1.1]** | **2.3[2.7;4.4]** |
|  | **LECT2** | **leukocyte cell-derived chemotaxin 2** | **-1.2[-1.5;-1.2]** | **-1.3[-1.7;-1.2]** | **-1.3[-2.5;-1.6]** | **-1.0[-1.6;-1.2]** | **-1.3[-3.2;-1.2]** | **-1.2[-2.1;-1.1]** | **-1.1[-1.6;1.0]** | **-2.3[-6.7;-1.4]** | **-6.0[-15.5;-4.9]** | **1.3[-1.2;1.4]** |
| Miscellanous | **CD14** | **CD14 molecule** | **1.5[1.3;2.2]** | **1.9[1.5;2.5]** | **1.9[1.9;2.5]** | **1.9[1.7;3.0]** | **2.4[2.0;4.1]** | **2.4[1.9;3.6]** | **1.4[1.2;2.0]** | **1.5[1.4;2.1]** | **-1.2[-2.7;1.6]** | **1.5[-1.1;2.1]** |
|  | **IRF7** | **interferon regulatory factor 7** | **1.4[1.1;1.8]** | **1.7[1.1;2.3]** | **1.4[1.1;2.1]** | **1.4[-1.1;2.6]** | **1.6[-1.1;2.5]** | **1.9[1.4;2.5]** | **1.7[1.2;3.5]** | **2.3[1.7;3.4]** | **2.0[1.3;4.0]** | **1.7[-1.1;3.6]** |
|  | **SGK2** | **serum/glucocorticoid regulated kinase 2** | **1.8[1.5;2.8]** | **2.0[1.8;3.2]** | **2.1[2.2;3.9]** | **2.0[2.4;4.1]** | **2.6[2.5;4.5]** | **1.3[-1.2;3.3]** | **2.1[2.1;2.9]** | **2.4[2.6;3.3]** | **1.2[-2.6;4.0]** | **1.9[1.5;3.2]** |
